# Supplementary material for: Nurse home visiting to improve child and maternal outcomes: 5-year follow-up of an Australian randomised controlled trial
Source: PLoS One. 2022 Nov 28;17(11):e0277773. doi: 10.1371/journal.pone.0277773 (PMC9704648; doi:10.1371/journal.pone.0277773)
Supplement: S1 Table — (DOCX) [file pone.0277773.s001.docx]

**S1 Table: Baseline characteristics according to follow-up status (i.e., retained or lost to follow-up) at child age 4 years.**

|  | **Intervention** | | |  | **Control** | | |  |  |
| --- | --- | --- | --- | --- | --- | --- | --- | --- | --- |
| **Baseline characteristics (pregnancy)** | **Allocated**  **(N=363)** | **Retained**  **(N=248)** | **Lost**  **(N=115)** |  | **Allocated**  **(N=359)** | **Retained**  **(N=217)** | **Lost**  **(N=142)** |  | **p-value** ^a^ |
| *Mother* |  |  |  |  |  |  |  |  |  |
| Age (years), mean (SD) | 27·5 (6·1) | 27·7 (6·0) | 27·0 (6·4) |  | 27·8 (6·4) | 28·5 (6·3) | 26·8 (6·3) |  | 0·14 |
| DASS Depression, mean (SD) | 3·1 (3·6) | 3·1 (3·5) | 3·1 (3·9) |  | 2·9 (3·3) | 2·9 (3·2) | 2·8 (3·4) |  | 0·60 |
| DASS Anxiety, mean (SD) | 3·6 (3·5) | 3·5 (3·5) | 3·8 (3·5) |  | 3·4 (3·3) | 3·3 (3·2) | 3·6 (3·5) |  | 0·53 |
| DASS Stress, mean (SD) | 5·5 (4·3) | 5·4 (4·0) | 5·8 (4·9) |  | 5·4 (4·0) | 5·5 (4·0) | 5·1 (4·2) |  | 0·68 |
| DASS Depression, >85th percentile score | 64 (17·6) | 43 (17·3) | 21 (18·3) |  | 57 (15·9) | 31 (14·3) | 26 (18·3) |  | 0·37 |
| DASS Anxiety, >85th percentile score | 157 (43·3) | 103 (41·5) | 54 (47·0) |  | 149 (41·5) | 84 (38·7) | 65 (45·8) |  | 0·54 |
| DASS Stress, >85th percentile score | 73 (20·1) | 47 (19·0) | 26 (22·6) |  | 68 (18·9) | 44 (20·3) | 24 (16·9) |  | 0·72 |
| Education status |  |  |  |  |  |  |  |  | 0·56 |
| Did not complete high school | 80 (24·8) | 49 (21·8) | 31 (32·0) |  | 82 (25·3) | 52 (26·1) | 30 (24·0) |  | NA |
| Completed high school / vocational training | 208 (64·6) | 149 (66·2) | 59 (60·8) |  | 207 (63·9) | 126 (63·3) | 81 (64·8) |  | NA |
| Completed a university degree | 34 (10·6) | 27 (12·0) | 7 (7·2) |  | 35 (10·8) | 21 (10·6) | 14 (11·2) |  | NA |
| Marital status |  |  |  |  |  |  |  |  | 0·41 |
| Single / not living with partner | 103 (28·4) | 69 (27·8) | 34 (29·6) |  | 92 (25·6) | 50 (23·0) | 42 (29·6) |  | NA |
| Married / living with partner | 253 (69·7) | 174 (70·2) | 79 (68·7) |  | 260 (72·4) | 164 (75·6) | 96 (67·6) |  | NA |
| Separated / divorced | 7 (1·9) | 5 (2·0) | 2 (1·7) |  | 7 (2·0) | 3 (1·4) | 4 (2·8) |  | NA |
| Currently unemployed | 239 (65·8) | 155 (62·5) | 84 (73·0) |  | 239 (66·6) | 134 (61·8) | 105 (73·9) |  | 0·87 |
| Family income from benefit or pension | 159 (43·8) | 102 (41·1) | 57 (49·6) |  | 150 (41·8) | 85 (39·2) | 65 (45·8) |  | 0·67 |
| Ever had a drug problem | 51 (14·1) | 30 (12·2) | 21 (18·3) |  | 60 (16·9) | 25 (11·6) | 35 (25·0) |  | 0·85 |
| Experienced family violence in past year | 44 (12·2) | 25 (10·2) | 19 (16·7) |  | 41 (11·5) | 23 (10·7) | 18 (12·8) |  | 0·85 |
| Total adversity risk count (from screening), mean (SD) | 3·1 (1·3) | 3·0 (1·2) | 3·5 (1·4) |  | 3·2 (1·2) | 3·2 (1·2) | 3·3 (1·2) |  | 0·95 |
| *Child* |  |  |  |  |  |  |  |  |  |
| First child | 135 (37·2) | 96 (38·7) | 39 (33·9) |  | 131 (36·5) | 75 (34·6) | 56 (39·4) |  | 0·36 |
| Female | 191 (54·4) | 141 (56·9) | 50 (48·5) |  | 154 (44·6) | 103 (47·5) | 51 (39·8) |  | 0·04 |
| *Family* |  |  |  |  |  |  |  |  |  |
| SEIFA Index of Social Disadvantage Quintile |  |  |  |  |  |  |  |  | 0·62 |
| 1 (most disadvantaged) | 157 (44·5) | 107 (44·0) | 50 (45·5) |  | 139 (40·2) | 84 (40·0) | 55 (40·4) |  | NA |
| 2 | 27 (7·7) | 16 (6·6) | 11 (10·0) |  | 30 (8·7) | 19 (9·1) | 11 (8·1) |  | NA |
| 3 | 132 (37·4) | 96 (39·5) | 36 (32·7) |  | 132 (38·2) | 81 (38·6) | 51 (37·5) |  | NA |
| 4 | 28 (7·9) | 16 (6·6) | 12 (10·9) |  | 32 (9·3) | 20 (9·5) | 12 (8·8) |  | NA |
| 5 (least disadvantaged) | 9 (2·6) | 8 (3·3) | 1 (0·9) |  | 13 (3·8) | 6 (2·9) | 7 (5·2) |  | NA |
| Language other than English | 29 (8·1) | 18 (7·4) | 11 (9·7) |  | 34 (9·7) | 18 (8·4) | 16 (11·7) |  | 0·67 |

^a^ p-value for chi-square tests (categorical measures) and t-tests (continuous measures) comparing those retained in the intervention and usual care groups.

All values are percentages, except where otherwise stated.

DASS= Depression, Anxiety, Stress Scale; SD=Standard Deviation; SEIFA=Socioeconomic Indexes for Areas Index of Relative Disadvantage
