# Supplementary material for: Nurse home visiting to improve child and maternal outcomes: 5-year follow-up of an Australian randomised controlled trial
Source: PLoS One. 2022 Nov 28;17(11):e0277773. doi: 10.1371/journal.pone.0277773 (PMC9704648; doi:10.1371/journal.pone.0277773)
Supplement: S2 Table — (DOCX) [file pone.0277773.s002.docx]

**S2 Table: Results of adjusted regression analyses comparing the two trial arms on child outcomes at age 4 and 5 years, using complete cases data.**

|  |  | **Descriptive statistics** | | | | **Comparative statistic: I compared to C (95% CI)** | | | | |
| --- | --- | --- | --- | --- | --- | --- | --- | --- | --- | --- |
| **Outcome** | **Child age** | **Intervention (I)** | | **Control (C)** | | **Adjusted** | | | **Effect Size** | **95% CI** |
|  |  | **N** | **Summary ^a^** | **N** | **Summary** ^a^ | **Statistic** ^d^ | **95% CI** | **P** |  |  |
| *Child Language and Learning* |  |  |  |  |  |  |  |  |  |  |
| CELF Sentence Structure | 4y | 226 | 8·45 (3·31) | 199 | 8·14 (3·65) | 0·30 | -0·32 to 0·93 | 0·33 | 0·09 | -0·09 to 0·27 |
|  | 5y | 208 | 9·34 (3·20) | 177 | 8·73 (3·27) | 0·68 | 0·01 to 1·35 | 0·05 | 0·21 | 0·00 to 0·42 |
| CELF Word Structure | 4y | 220 | 8·49 (2·99) | 193 | 8·45 (3·48) | -0·08 | -0·55 to 0·40 | 0·74 | -0·02 | -0·17 to 0·12 |
|  | 5y | 205 | 9·09 (3·16) | 175 | 8·46 (3·39) | 0·50 | 0·01 to 0·99 | 0·04 | 0·15 | 0·00 to 0·30 |
| CELF Expressive Vocabulary | 4y | 220 | 8·92 (2·94) | 196 | 8·64 (3·12) | 0·20 | -0·26 to 0·66 | 0·37 | 0·07 | -0·08 to 0·22 |
|  | 5y | 203 | 8·70 (2·54) | 175 | 8·41 (3·03) | 0·18 | -0·23 to 0·58 | 0·38 | 0·06 | -0·08 to 0·21 |
| CELF Core Language | 4y | 220 | 92·19 (15·77) | 192 | 90·88 (17·66) | 0·87 | -1·59 to 3·32 | 0·47 | 0·05 | -0·10 to 0·20 |
|  | 5y | 203 | 94·74 (14·49) | 173 | 91·84 (15·91) | 2·60 | 0·21 to 4·99 | 0·03 | 0·17 | 0·01 to 0·33 |
| SEAPART Syllable Clapping | 4y | 212 | 5·42 (3·24) | 189 | 4·98 (3·35) | 0·41 | -0·23 to 1·05 | 0·20 | 0·12 | -0·07 to 0·32 |
|  | 5y | 196 | 7·94 (2·75) | 167 | 7·66 (2·85) | 0·01 | -0·55 to 0·56 | 0·98 | 0·00 | -0·20 to 0·20 |
| SEAPART Syllable Isolation | 4y | 210 | 0·54 (1·20) | 186 | 0·47 (1·13) | 0·02 | -0·24 to 0·27 | 0·88 | 0·02 | -0·20 to 0·23 |
|  | 5y | 194 | 2·87 (2·80) | 164 | 2·48 (2·63) | 0·19 | -0·13 to 0·51 | 0·24 | 0·07 | -0·05 to 0·19 |
| SEAPART First Sound Identification | 4y | 202 | 1·62 (2·90) | 177 | 1·50 (2·79) | 0·25 | -0·41 to 0·90 | 0·45 | 0·09 | -0·14 to 0·32 |
|  | 5y | 193 | 5·39 (4·02) | 160 | 4·94 (4·21) | 0·37 | -0·30 to 1·05 | 0·27 | 0·09 | -0·07 to 0·26 |
| SEAPART Letter Identification | 4y | 206 | 1·42 (2·31) | 180 | 1·32 (2·33) | 0·08 | -0·33 to 0·49 | 0·69 | 0·03 | -0·14 to 0·21 |
|  | 5y | 196 | 4·07 (3·33) | 162 | 3·78 (3·55) | 0·01 | -0·62 to 0·65 | 0·97 | 0·00 | -0·18 to 0·19 |
| SEAPART Name Writing | 4y | 210 | 0·88 (1·72) | 181 | 0·85 (1·64) | 0·06 | -0·26 to 0·39 | 0·70 | 0·04 | -0·16 to 0·23 |
|  | 5y | 196 | 3·91 (2·06) | 162 | 3·43 (2·17) | 0·32 | -0·06 to 0·69 | 0·09 | 0·15 | -0·03 to 0·33 |
| SEAPART Rhyme Detection | 4y | 196 | 2·43 (1·94) | 162 | 2·59 (2·33) | -0·20 | -0·58 to 0·17 | 0·27 | -0·10 | -0·27 to 0·08 |
|  | 5y | 189 | 2·63 (1·19) | 159 | 2·58 (1·25) | 0·03 | -0·26 to 0·32 | 0·83 | 0·02 | -0·21 to 0·26 |
| SEAPART Total Score | 4y | 195 | 10·21 (7·79) | 166 | 9·49 (7·75) | 0·70 | -1·17 to 2·56 | 0·45 | 0·09 | -0·15 to 0·33 |
|  | 5y | 188 | 30·40 (5·46) | 149 | 28·94 (5·85) | 1·20 | 0·34 to 2·06 | 0·01 | 0·21 | 0·06 to 0·36 |
| NIH Toolbox Flanker Inhibitory Control & Attention | 4y | 210 | 96·36 (13·93) | 184 | 93·67 (15·45) | 2·75 | -0·23 to 5·73 | 0·07 | 0·19 | -0·02 to 0·39 |
|  | 5y | 206 | 98·29 (12·57) | 173 | 96·08 (13·29) | 2·83 | -0·35 to 6·00 | 0·08 | 0·22 | -0·03 to 0·46 |
| NIH Toolbox Dimensional Change Card Sort | 4y | 198 | 98·11 (11·64) | 172 | 96·23 (13·15) | 1·82 | -0·87 to 4·50 | 0·18 | 0·15 | -0·07 to 0·36 |
|  | 5y | 205 | 97·47 (13·14) | 166 | 97·02 (12·38) | -0·26 | -3·34 to 2·81 | 0·86 | -0·02 | -0·26 to 0·22 |
| *Child Health and Mental health* |  |  |  |  |  |  |  |  |  |  |
| SDQ Externalizing (reverse) | 4y | 243 | 12·49 (3·95) | 212 | 11·94 (3·64) | 0·37 | -0·17 to 0·90 | 0·17 | 0·10 | -0·04 to 0·24 |
|  | 5y | 218 | 13·71 (3·52) | 195 | 12·87 (3·78) | 0·59 | 0·02 to 1·16 | 0·04 | 0·16 | 0·01 to 0·32 |
| SDQ Internalizing (reverse) | 4y | 243 | 16·31 (2·70) | 213 | 16·00 (2·96) | 0·23 | -0·10 to 0·56 | 0·17 | 0·08 | -0·04 to 0·20 |
|  | 5y | 218 | 16·69 (2·82) | 194 | 16·08 (3·04) | 0·59 | 0·13 to 1·06 | 0·01 | 0·20 | 0·04 to 0·36 |
| SDQ Total Behavior (reverse) | 4y | 243 | 28·81 (5·83) | 212 | 27·94 (5·56) | 0·60 | -0·02 to 1·22 | 0·06 | 0·10 | -0·00 to 0·21 |
|  | 5y | 218 | 30·40 (5·46) | 194 | 28·94 (5·85) | 1·20 | 0·34 to 2·06 | 0·01 | 0·21 | 0·06 to 0·36 |
| SSIS Communication | 5y | 217 | 16·35 (3·21) | 189 | 16·08 (3·28) | 0·42 | -0·08 to 0·92 | 0·10 | 0·13 | -0·03 to 0·28 |
| SSIS Cooperation | 5y | 217 | 12·68 (3·22) | 189 | 12·58 (3·17) | 0·16 | -0·40 to 0·73 | 0·55 | 0·05 | -0·12 to 0·23 |
| SSIS Assertion | 5y | 217 | 15·00 (3·00) | 189 | 14·69 (3·25) | 0·47 | -0·03 to 0·96 | 0·06 | 0·15 | -0·01 to 0·31 |
| SSIS Responsibility | 5y | 217 | 12·34 (3·13) | 189 | 11·95 (3·34) | 0·39 | -0·27 to 1·04 | 0·23 | 0·12 | -0·08 to 0·32 |
| SSIS Empathy | 5y | 217 | 13·71 (3·37) | 189 | 13·60 (3·28) | 0·25 | -0·33 to 0·82 | 0·38 | 0·07 | -0·10 to 0·25 |
| SSIS Engagement | 5y | 217 | 15·92 (3·66) | 189 | 15·50 (3·74) | 0·57 | -0·02 to 1·16 | 0·06 | 0·15 | -0·01 to 0·31 |
| SSIS Self-control | 5y | 217 | 11·51 (3·77) | 189 | 10·95 (4·08) | 0·77 | 0·20 to 1·35 | 0·01 | 0·20 | 0·05 to 0·34 |
| SSIS Total | 5y | 217 | 97·52 (19·48) | 189 | 95·36 (20·13) | 3·03 | -0·26 to 6·32 | 0·07 | 0·15 | -0·01 to 0·32 |
| PedsQL Physical Wellbeing | 4y | 242 | 87·59 (13·86) | 215 | 87·11 (14·26) | 0·44 | -2·02 to 2·90 | 0·71 | 0·03 | -0·14 to 0·21 |
|  | 5y | 219 | 88·12 (13·38) | 197 | 87·26 (12·43) | 0·83 | -1·59 to 3·26 | 0·49 | 0·06 | -0·12 to 0·25 |
| PedsQL Socioemotional Wellbeing | 4y | 243 | 82·34 (13·23) | 215 | 80·26 (13·72) | 1·62 | -0·58 to 3·83 | 0·14 | 0·12 | -0·04 to 0·28 |
|  | 5y | 219 | 82·44 (13·60) | 197 | 80·31 (14·45) | 1·53 | -0·48 to 3·55 | 0·13 | 0·11 | -0·03 to 0·25 |
| Stress (hair cortisol, pg/mg ^b^) | 4y | 146 | -1·72 (0·96) | 118 | -1·68 (0·72) | -0·08 | -0·29 to 0·13 | 0·44 | -0·09 | -0·33 to 0·15 |
|  | 5y | 116 | -1·22 (0·88) | 89 | -1·14 (0·94) | -0·15 | -0·42 to 0·12 | 0·26 | -0·17 | -0·47 to 0·13 |
| No dental caries^c^ | 4y | 214 | 173 (80·84) | 194 | 159 (81·96) | 1·04 | 0·60 to 1·80 | 0·90 | NA | NA |
|  | 5y | 206 | 179 (86·89) | 174 | 148 (85·06) | 1·30 | 0·66 to 2·55 | 0·45 | NA | NA |
| Not overweight/obese ^c^ | 4y | 223 | 170 (76·23) | 196 | 146 (74·49) | 0·98 | 0·74 to 1·29 | 0·89 | NA | NA |
|  | 5y | 210 | 153 (72·86) | 176 | 131 (74·43) | 0·79 | 0·49 to 1·28 | 0·34 | NA | NA |

I= Intervention; C= Control; CI= Confidence Interval; CELF= Clinical Evaluation of Language Fundamentals; SEAPART= School Entry Alphabetic and Phonological Awareness Readiness Test; NIH= SDQ= Strengths and Difficulties Questionnaire; PedsQL= Pediatric Quality of Life Inventory; DASS= Depression Anxiety and Stress Scales; AQoL= Adult Quality of Life.

^a^ Summary statistics are mean except where specified as dichotomous.

^b^ Hair cortisol is log transformed and negativized, so that higher values indicate lower cortisol.

^c^ Outcome is dichotomous (%), comparative statistics is odds ratio (OR).

^d^ The comparative statistic is mean difference for continuous outcomes (intervention minus control) and odds ratio for dichotomous outcomes (the risk of outcome for those receiving the intervention compared with receiving usual care).
