# Supplementary material for: Nurse home visiting to improve child and maternal outcomes: 5-year follow-up of an Australian randomised controlled trial
Source: PLoS One. 2022 Nov 28;17(11):e0277773. doi: 10.1371/journal.pone.0277773 (PMC9704648; doi:10.1371/journal.pone.0277773)
Supplement: S3 Table — (DOCX) [file pone.0277773.s003.docx]

**S3 Table: Results of adjusted regression analyses comparing the two trial arms on maternal outcomes at child ages 4 and 5 years, using complete cases data.**

|  |  | Descriptive statistics | | | | Comparative statistic: I compared to C (95% CI) | | | | |
| --- | --- | --- | --- | --- | --- | --- | --- | --- | --- | --- |
| Outcome | **Child age** | **Intervention (I)** | |  | **Control (C)** | Adjusted | | | Effect Size | 95% CI |
|  |  | **N** | **Summary** ^a^ | **N** | **Summary** ^a^ | **Statistic** ^d^ | **95% CI** | **p** |  |  |
| *Parenting and Family Relationships* | | |  |  |  |  |  |  |  |  |
| Warm parenting | 4y | 242 | 4·67 (0·48) | 211 | 4·60 (0·47) | 0·09 | 0·01 to 0·17 | 0·03 | 0·19 | 0·02 to 0·35 |
|  | 5y | 214 | 4·74 (0·39) | 185 | 4·64 (0·48) | 0·08 | 0·00 to 0·15 | 0·05 | 0·17 | 0·00 to 0·35 |
| Hostile parenting (reverse) | 4y | 245 | 7·89 (1·61) | 216 | 7·72 (1·52) | 0·28 | 0·03 to 0·52 | 0·03 | 0·18 | 0·02 to 0·33 |
|  | 5y | 219 | 7·72 (1·67) | 196 | 7·67 (1·59) | 0·22 | -0·04 to 0·48 | 0·10 | 0·13 | -0·03 to 0·29 |
| Parenting efficacy | 4y | 242 | 8·16 (1·48) | 214 | 7·96 (1·36) | 0·23 | -0·01 to 0·48 | 0·06 | 0·16 | -0·01 to 0·33 |
|  | 5y | 219 | 8·33 (1·51) | 191 | 8·00 (1·52) | 0·28 | 0·07 to 0·48 | 0·01 | 0·18 | 0·05 to 0·32 |
| Parent-child closeness | 4y | 241 | 33·34 (2·98) | 210 | 33·17 (2·77) | 0·20 | -0·32 to 0·71 | 0·44 | 0·07 | -0·11 to 0·25 |
|  | 5y | 215 | 33·35 (2·63) | 193 | 33·07 (2·51) | 0·31 | -0·03 to 0·65 | 0·07 | 0·12 | -0·01 to 0·25 |
| Parent-child conflict (reverse) | 4y | 239 | 21·93 (6·98) | 212 | 20·97 (6·48) | 0·82 | -0·34 to 1·98 | 0·16 | 0·12 | -0·05 to 0·29 |
|  | 5y | 216 | 21·96 (6·82) | 193 | 21·09 (6·48) | 0·73 | -0·33 to 1·79 | 0·17 | 0·11 | -0·05 to 0·27 |
| Regular meal times ^b^ | 4y | 242 | 224 (92·56) | 214 | 191 (89·25) | 1·65 | 0·84 to 3·24 | 0·14 | NA | NA |
|  | 5y | 220 | 211 (95·91) | 197 | 176 (89·34) | 3·40 | 1·86 to 6·20 | <0·001 | NA | NA |
| Regular bedtime^b^ | 4y | 243 | 205 (84·36) | 213 | 171 (80·28) | 1·24 | 0·72 to 2·14 | 0·43 | NA | NA |
|  | 5y | 220 | 200 (90·91) | 197 | 160 (81·22) | 2·86 | 1·63 to 5·04 | <0·001 | NA | NA |
| Regular bed routine ^b^ | 4y | 242 | 198 (81·82) | 214 | 163 (76·17) | 1·48 | 0·99 to 2·21 | 0·06 | NA | NA |
|  | 5y | 218 | 182 (83·49) | 197 | 154 (78·17) | 1·67 | 1·18 to 2·35 | 0·003 | NA | NA |
| Child-parent relationship (very good/excellent) ^b^ | 4y | 246 | 223 (90·65) | 214 | 198 (92·52) | 0·96 | 0·46 to 2·02 | 0·92 | NA | NA |
|  | 5y | 221 | 203 (91·86) | 194 | 182 (93·81) | 0·53 | 0·26 to 1·06 | 0·07 | NA | NA |
| No reported emotional abuse ^b^ | 4y | 164 | 116 (70·73) | 141 | 99 (70·21) | 0·89 | 0·56 to 1·42 | 0·64 | NA | NA |
|  | 5y | 131 | 105 (80·15) | 120 | 81 (67·50) | 1·82 | 0·98 to 3·38 | 0·06 | NA | NA |
| *Maternal Mental Health and Wellbeing* |  |  |  |  |  |  |  |  |  |  |
| DASS - Depression (reverse) | 4y | 247 | 18·29 (3·84) | 213 | 17·58 (4·02) | 0·66 | 0·05 to 1·27 | 0·03 | 0·17 | 0·01 to 0·32 |
|  | 5y | 223 | 18·16 (3·49) | 197 | 17·75 (3·60) | 0·29 | -0·47 to 1·04 | 0·44 | 0·08 | -0·13 to 0·29 |
| DASS - Anxiety (reverse) | 4y | 245 | 18·53 (3·46) | 215 | 18·33 (3·07) | 0·27 | -0·13 to 0·68 | 0·17 | 0·08 | -0·04 to 0·21 |
|  | 5y | 221 | 18·30 (3·37) | 197 | 18·10 (3·50) | 0·04 | -0·68 to 0·76 | 0·90 | 0·01 | -0·20 to 0·22 |
| DASS - Stress (reverse) | 4y | 246 | 15·91 (4·20) | 214 | 15·49 (4·34) | 0·42 | -0·33 to 1·18 | 0·26 | 0·10 | -0·08 to 0·28 |
|  | 5y | 224 | 15·71 (3·99) | 197 | 15·24 (4·33) | 0·37 | -0·46 to 1·19 | 0·37 | 0·09 | -0·11 to 0·29 |
| DASS - Overall (reverse) | 4y | 245 | 52·72 (10·73) | 213 | 51·38 (10·44) | 1·37 | -0·27 to 3·00 | 0·10 | 0·13 | -0·03 to 0·28 |
|  | 5y | 220 | 52·23 (9·98) | 197 | 51·09 (10·32) | 0·77 | -1·41 to 2·95 | 0·47 | 0·08 | -0·14 to 0·29 |
| Personal Wellbeing Index | 4y | 242 | 59·78 (11·97) | 209 | 56·80 (12·09) | 2·57 | 1·27 to 3·87 | <0·001 | 0·21 | 0·11 to 0·32 |
|  | 5y | 218 | 59·89 (11·81) | 193 | 56·71 (12·71) | 2·67 | 0·41 to 4·92 | 0·02 | 0·22 | 0·03 to 0·40 |
| AQoL | 4y | 239 | 0·70 (0·19) | 212 | 0·67 (0·19) | 0·03 | 0·00 to 0·07 | 0·04 | 0·17 | 0·01 to 0·34 |
|  | 5y | 220 | 0·70 (0·19) | 197 | 0·67 (0·19) | 0·02 | -0·01 to 0·05 | 0·21 | 0·10 | -0·06 to 0·26 |
| Stress (hair cortisol, pg/mg)^c^ | 4y | 174 | -1·38 (0·69) | 152 | -1·47 (0·69) | 0·01 | -0·14 to 0·15 | 0·90 | 0·01 | -0·20 to 0·22 |
|  | 5y | 127 | -1·11 (0·69) | 116 | -1·23 (0·88) | 0·08 | -0·11 to 0·26 | 0·42 | 0·10 | -0·14 to 0·34 |
| DASS - Depression (reverse, <15th percentile) ^b^ | 4y | 247 | 218 (88·26) | 213 | 177 (83·10) | 1·58 | 0·98 to 2·54 | 0·06 | NA | NA |
|  | 5y | 223 | 186 (83·41) | 197 | 158 (80·20) | 1·24 | 0·82 to 1·87 | 0·31 | NA | NA |
| DASS - Anxiety (reverse, <15th percentile) ^b^ | 4y | 245 | 195 (79·59) | 215 | 170 (79·07) | 1·06 | 0·80 to 1·42 | 0·67 | NA | NA |
|  | 5y | 221 | 167 (75·57) | 197 | 150 (76·14) | 0·86 | 0·59 to 1·26 | 0·45 | NA | NA |
| DASS - Stress (reverse, <15th percentile) ^b^ | 4y | 246 | 216 (87·80) | 214 | 176 (82·24) | 1·68 | 1·00 to 2·84 | 0·05 | NA | NA |
|  | 5y | 224 | 191 (85·27) | 197 | 156 (79·19) | 1·68 | 0·91 to 3·12 | 0·10 | NA | NA |
| Very good/excellent health ^b^ | 4y | 243 | 75 (30·86) | 216 | 52 (24·07) | 1·50 | 1·13 to 1·98 | 0·005 | NA | NA |
|  | 5y | 220 | 67 (30·45) | 196 | 45 (22·96) | 1·42 | 1·00 to 2·00 | 0·05 | NA | NA |
| Currently employed ^b^ | 4y | 247 | 104 (42·11) | 216 | 82 (37·96) | 1·10 | 0·79 to 1·53 | 0·59 | NA | NA |
|  | 5y | 224 | 103 (45·98) | 199 | 88 (44·22) | 0·87 | 0·65 to 1·17 | 0·36 | NA | NA |
| Currently studying ^b^ | 4y | 246 | 39 (15·85) | 216 | 37 (17·13) | 0·96 | 0·62 to 1·50 | 0·86 | NA | NA |
|  | 5y | 219 | 32 (14·61) | 197 | 41 (20·81) | 0·60 | 0·40 to 0·90 | 0·01 | NA | NA |
| Planning future study ^b^ | 4y | 245 | 119 (48·57) | 216 | 124 (57·41) | 0·81 | 0·57 to 1·15 | 0·24 | NA | NA |
|  | 5y | 224 | 125 (55·80) | 200 | 115 (57·50) | 1·04 | 0·70 to 1·55 | 0·84 | NA | NA |
| Self-efficacy ^b^ | 4y | 243 | 201 (82·72) | 203 | 153 (75·37) | 1·47 | 1·02 to 2·12 | 0·04 | NA | NA |
|  | 5y | 223 | 177 (79·37) | 194 | 138 (71·13) | 1·76 | 1·21 to 2·57 | 0·003 | NA | NA |
| Doesn’t smoke ^b^ | 4y | 244 | 163 (66·80) | 214 | 148 (69·16) | 0·81 | 0·53 to 1·24 | 0·33 | NA | NA |
|  | 5y | 216 | 142 (65·74) | 193 | 130 (67·36) | 0·81 | 0·54 to 1·20 | 0·29 | NA | NA |

I= Intervention; C= Control; CI= Confidence Interval; CELF= Clinical Evaluation of Language Fundamentals; SEAPART= School Entry Alphabetic and Phonological Awareness Readiness Test; NIH= SDQ= Strengths and Difficulties Questionnaire; PedsQL= Pediatric Quality of Life Inventory; DASS= Depression Anxiety and Stress Scales; AQoL= Adult Quality of Life.

^a^ Summary statistics are mean except where specified as dichotomous.

^b^ Outcome is dichotomous (%).

^c^ Hair cortisol is log transformed and negativized, so that higher values indicate lower cortisol.

^d^ The comparative statistic is mean difference for continuous outcomes (intervention minus control) and odds ratio for dichotomous outcomes (the risk of receiving the intervention compared with receiving usual care)
