# Supplementary material for: Nurse home visiting to improve child and maternal outcomes: 5-year follow-up of an Australian randomised controlled trial
Source: PLoS One. 2022 Nov 28;17(11):e0277773. doi: 10.1371/journal.pone.0277773 (PMC9704648; doi:10.1371/journal.pone.0277773)
Supplement: S2 File — (PDF) [file pone.0277773.s006.pdf]

<STUDY IDENTIFIER>

right@home

<FULL STUDY TITLE>

right@home: a randomised controlled trial of sustained, nurse home visiting measuring the benefit on parenting and the home environment when offered to vulnerable Australian mothers from the antenatal period to child age 2 years.

<LAY STUDY TITLE>

right@home: A national sustained nurse home visiting trial to promote family wellbeing and child development

<VERSION #, DATE>

Version 4, 12 March 2013

CONFIDENTIAL

This document is confidential and the property of Murdoch Childrens Research Institute. No part of it may be transmitted, reproduced, published, or used without prior written authorization from the institution.

STATEMENT OF COMPLIANCE

This document is a protocol for a clinical research study. The study will be conducted in compliance with all stipulations of this protocol, the conditions of ethics committee approval, the NHMRC National Statement on Ethical Conduct in Human Research (2007) and the Note for Guidance on Good Clinical Practice (CPMP/ICH-135/95).

TABLE OF CONTENTS

Page

STATEMENT OF COMPLIANCE 1
TABLE OF CONTENTS 2
GLOSSARY OF ABBREVIATIONS 5
1. INVESTIGATORS AND FACILITIES 6
2. INTRODUCTION AND BACKGROUND 12
2.1 Research Question 13
2.2 Rationale for Current Study 13
3. STUDY OBJECTIVES 14
3.1 Primary Objectives 14
3.2 Secondary Objectives 14
4. STUDY DESIGN 15
4.1 Type of Study 15
4.2 Study Design Diagram 17
4.3 Number of Subjects 19
4.4 Expected Duration of Study 19
4.5 Primary and Secondary Outcome Measures 19
5. STUDY TREATMENTS 21
5.1 Treatment Arms 21
5.1.1 Description 21
5.1.2 Dosage and Route of Administration 23
5.2 Measurement of subject compliance 24
5.3 Excluded medications and treatments 24
6. SUBJECT ENROLLMENT AND RANDOMISATION 24
6.1 Recruitment 24
6.2 Eligibility Criteria 27
6.3 Randomisation Procedures 30
6.4 Blinding Arrangements 31
6.5 Breaking Of The Study Blind 31
6.7 Trial Closure 32
6.8 Continuation of Therapy 33
7. STUDY VISITS AND PROCEDURES SCHEDULE 33
8. CLINICAL AND LABORATORY ASSESSMENTS 36
9. ADVERSE EVENT REPORTING 37
9.1 Definitions 37
9.3 Eliciting Adverse Event Information 39
9.4 Serious Adverse Event Reporting 39
10. STATISTICAL METHODS 40
10.1 Sample Size Estimation 40

10.2 Population to be analysed 41
10.3 Statistical Analysis Plan 41
10.4 Interim Analyses 42
11. DATA MANAGEMENT 42
12. ADMINISTRATIVE ASPECTS 43
13. USE OF DATA AND PUBLICATIONS POLICY 44
14. LIST OF ATTACHED APPENDICES 44
15. REFERENCES 46

PROTOCOL SYNOPSIS

Title	**right@home**: a randomised controlled trial of sustained, nurse home visiting measuring the benefit on parenting and the home environment when offered to vulnerable Australian mothers from the antenatal period to child age 2 years.
Objectives	At child age 2 years, compared with the control group, intervention mothers will demonstrate improved:<br>(1) Parent care, i.e. the parent's ability to provide a consistent, regular and supportive environment for their child;<br>(2) Parent responsivity, i.e. the parent's ability to tune in to their child's needs and to respond appropriately; and<br>(3) Supportive home environment, i.e. building a strong home learning environment through structured developmental promotion activities focusing on language
Design	Randomised controlled trial
Outcomes	Primary outcomes:<br>(1) Parent care,<br>(2) Parent responsivity, and<br>(3) Home Environment.<br><br>Secondary Outcomes:<br>1. Maternal: Pregnancy outcomes, Quality of Life, Maternal mental health, Parent wellbeing, General Health, Parenting self-efficacy, Health service use and Health literacy<br>2. Child: Mental and general health, and Language<br>3. Sibling: Mental health/behaviour<br>4. Nurse: Use and knowledge of evidence-based strategies
Study Duration	5 years: January 2013 to December 2017
Interventions	At least 25 home visits (actual number of visits determined by need) by a right@home nurse. Nurses are maternal and child (MCH) nurses who will be trained to deliver the following:<br>a) Antenatal and postnatal care in accordance with the MCH and MECSH guidelines. The content of home visits will focus on the three primary outcomes listed above, and will be individually tailored to the mother's needs, skills, strengths and capacity.<br>b) Postnatal Learning to Communicate program focussing on language promotion<br>c) Access to early childhood health services, volunteer home visiting services and family support services.<br>d) Group activities and community links including parenting group and walking group specifically for intervention families, and linking into community activities.
Number of subjects	n=800 pregnant women

Population	Vulnerable, pregnant Australian women, who have sufficient English proficiency to answer questions face-to-face. The inclusion criteria, based on the results of our pilot (RCH HREC 32771, Northern Health HREC P 20/12), are any two of the following 10 risk factors:<br>o Current smoking,<br>o Young pregnancy (<23 years old),<br>o No support (emotional, financial, practical) during pregnancy,<br>o Poor/fair/good general health (versus very good/excellent general health)<br>o Anxious mood,<br>o Not finishing high school,<br>o Not having a household income,<br>o A long-term illness,<br>o Living without another adult, and<br>o Not working previously.<br><br>Women will be excluded and/or discontinued if:<br>▪ Their child is removed from the home during the course of the research such that they are ineligible for the trial<br>▪ They are enrolled in the Victorian Department of Human Services 'Cradle to Kinder' research program<br>▪ They are enrolled in the Tasmanian Department of Health and Human Services CU@home program<br>▪ Do not comprehend the recruitment invitation (e.g. have an intellectual disability such that they are unable to consent to entering the study, or have insufficient English to complete face-to-face assessments, i.e. require an interpreter)<br>▪ Have no mechanism for contact, i.e. landline or mobile telephone, or email address<br>▪ Experience a critical event such as miscarriage, late termination pregnancy, stillbirth and neonatal death.

GLOSSARY OF ABBREVIATIONS

**ABBREVIATION**	**TERM**
ARACY	Australian Research Alliance for Children and Youth
AQoL	Assessment Quality of Life
CCCH	Centre for Community Child Health
CHETRE	Centre for Health Equity Training Research and Evaluation
CRF	Case Report Form
CtK	Cradle to Kinder

DASS	Depression, Anxiety, Stress Scale
DEECD	Department of Education and Early Childhood Development (Vic)
DHHS	Department of Health and Human Services (Tas)
ERG	Expert Reference Group
EPDS	Edinburgh Postnatal Depression Scale
GHQ	General Health Questionnaire
HOME	Home Observation for Measurement of the Environment
KAS	Key Age and Stage Visit (MCH)
LGA	Local Government Area
LSAC	Longitudinal Study of Australian Children
MCH	Maternal and Child Health
MCDI	MacArthur Communicative Developmental Inventory
MCRI	Murdoch Childrens Research Institute
MECSH	Maternal Early Childhood Sustained Home Visiting
PWI	Parenting Wellbeing Index
RCH	The Royal Children's Hospital, Melbourne, Victoria
RCT	Randomised Controlled Trial
SNHV	Sustained nurse home visiting
SAE	Serious Adverse Effect
SD	Standard Deviation
SDQ	Strengths and Difficulties Questionnaire
SUSAR	Suspected Unexpected Serious Adverse Reaction
UR	Unit Record

1. INVESTIGATORS AND FACILITIES

1.1 Study Location/s

This is a multi-state, multi-site trial, which will recruit families from the maternity hospitals that are aligned with corresponding local government areas (LGAs), both listed in Table 1 below. Families recruited from the hospital may live beyond the LGA borders but still be eligible for the trial.

There are two core elements underpinning site selection and recruitment. First, we have individual verbal/email agreements with maternity management at each of the listed sites for study participation. These sites are only involved with recruitment for the trial, and not the intervention or follow-up assessments. The choice of sites developed from extensive research into the suitability of birthing hospitals for the trial. Factors such as the birth rate and the proportion of women birthing

locally were taken into account when deciding which sites were most suitable for recruitment. Participation will, of course, be dependent on Ethics approval from the relevant HRECs.

Second, for any of the maternity hospitals, a local government area (LGA) is required to support the nurses to deliver the intervention, e.g. hire nurses, provide opportunities for training in the programme, and hire the support services including social work and Tier 2 services. Any hospital may serve a number of LGAs, and ARACY has been working with the local councils listed below to develop partnerships. These are formalised with a Memorandum of Understanding for each LGA.

Table 1: Participating sites and local councils

Hospital	Local Council
**Victoria**	
1. Dandenong Hospital<br>Address: Southern Health / David Street, Dandenong 3175.<br>Midwifery Unit Manager Allison Deering<br>Tel: 03 9554 8832, 0404 025 781<br>Email: allison.deering@southernhealth.org.au	Greater Dandenong City Council<br>Address: 397-405 Springvale Rd, SPRINGVALE 3171; Postal PO Box 200, DANDENONG 3175;<br>Tel 03 9239 5100, 03 9239 5100;<br>Fax 03 9239 5196, 03 9239 5196;<br>Email council@cgd.vic.gov.au;<br>Website www.greaterdandenong.com;<br>Chief Executive Officer Mr John Bennie
2. Frankston Hospital<br>Address: 2 Hastings Road, Frankston VIC 3199<br>Nurse Unit Manager: Della Attwood<br>Tel: 03 9784 8386, 9783 8324 (reception)<br>Email: dattwood@phcn.vic.gov.au<br>Acting Nurse Unit Manager: Kate Brown<br>Tel: 03 9784 7450<br>Email: katebrown@phcn.vic.gov.au	Frankston City Council<br>Address: Cnr Young & Davey Sts, FRANKSTON 3199; Postal PO Box 490, FRANKSTON 3199;<br>Tel 1300 322 322, 1300 322 322;<br>Fax 03 9784 1094, 03 9784 1094;<br>Email correspondence@frankston.vic.gov.au;<br>Website www.frankston.vic.gov.au;<br>Chief Executive Officer Ms Jane Homewood (acting)
3. Ballarat Hospital<br>Address: Drummond Street North Ballarat VIC 3350<br>Acting Director of Nursing: Joanne Gilbert, filling in for Director of Nursing, Women's & Children's Services and Clinical Nurse Consultants: Terri Antonio<br>Tel: 03 5320 4969, 0466 207 271<br>Email: joanneg@bhs.org.au / terria@bhs.org.au	Ballarat City Council<br>Address: 25 Armstrong Street South, BALLARAT 3350; Postal PO Box 655, BALLARAT 3353;<br>Tel 03 5320 5500, 03 5320 5500;<br>Fax 03 5320 5832, 03 5320 5832;<br>Email ballcity@ballarat.vic.gov.au;<br>Website www.ballarat.vic.gov.au;<br>Chief Executive Officer Mr Anthony Schinck
4. The Northern Hospital<br>Address: 185 Cooper Street Epping VIC 3076<br>Operations Director, Acute Women's & Children's Services: Michelle Morrow	Whittlesea City Council<br>Address: 25 Ferres Boulevard, SOUTH MORANG 3752; Postal Locked Bag 1, BUNDOORA MDC 3083;

Tel: 03 8405 8023, 0429 383 708<br>Email: Michelle.Morrow@nh.org.au	Tel 03 9217 2170, 03 9217 2170;<br>Fax 03 9217 2111, 03 9217 2111;<br>Email info@whittlesea.vic.gov.au;<br>Website www.whittlesea.vic.gov.au;<br>Chief Executive Officer Mr David Turnbull
**Tasmania**	
1. The Royal Hobart Hospital<br>Address: 48 Liverpool Street, Hobart, TAS 7000<br>Maternity Services and Women's Health Clinic Acting Nurse Unit Manager: Jodie Semmler, filling in for Nurse Unit Manager: Becky French<br>Tel: 03 6222 8299, 0438 457 030<br>Email: semmler@dhhs.tas.gov.au / becky.french@dhhs.tas.gov.au	Hobart City Council<br>Address: 16 Elizabeth St, Hobart, Tasmania 7001; Postal GPO Box 503 Hobart, Tasmania 7001;<br>Tel 03 6238 2711;<br>Email hcc@hobartcity.com.au;<br>Website http://www.hobartcity.com.au/Home
2. Launceston General Hospital<br>Address: Charles Street, Launceston, TAS 7250<br>Queen Victoria Maternity Unit Manager: Sue McBeath<br>Tel: 03 6348 8999<br>Email: sue.mcbeath@dhhs.tas.gov.au	Launceston City Council<br>Address: Customer Service Centre, Town Hall, St John Street, Launceston, Tasmania 7250; Postal PO Box 396 Launceston, Tasmania, 7250;<br>Tel 03 6323 3000;<br>Email council@launceston.tas.gov.au;<br>Website http://www.launceston.tas.gov.au/lcc/
3. The Northwest Regional Hospital<br>Address: 23 Brickport Road, Burnie, TAS 7370<br>North West Antenatal Services Manager & Outreach Maternity Services Co-ordinator: Alana Jarvis<br>Tel: 03 6432 6000<br>Email: alana.jarvis@healthecare.com.au	Burnie City Council<br>Address: 80 Wilson Street, Burnie, Tasmania DX 70210; Postal PO Box 973 Burnie, Tasmania 7320;<br>Tel 03 6430 5700;<br>Email burnie@burnie.net;<br>Website http://www.burnie.net/Home

1.2 Study Management and Governance

The governance structure is presented in Figure 1. This trial will be conducted by three partners: ARACY, CHETRE and CCCH (the Partnership). The trial itself consists of 2 main streams of work: the programme (intervention) element and the research (evaluation) element. Although the Partnership is responsible for the trial overall, the programme element will be coordinated and lead by a team comprising the Project manager, Registered Nurse, Postdoctoral Researchers and Research Assistants based at ARACY (ACT) and CHETRE (NSW). The research element of the trial will be coordinated and lead by a team based at the CCCH comprising the Principal Investigator, Project Manager, Paediatrician, Statistician, Health Economist and Research Assistants. ARACY will have overall project management responsibility to ensure that the two streams of work remain coordinated when necessary.

Qualified maternal and child health nurses will deliver the intervention. Trained research assistants will conduct the informed consent discussions and clinical assessments with participating women and children. The project coordinator (ARACY) and CHETRE will be responsible for hiring and training nurses. The research manager (CCCH) will be responsible for organising recruitment, participant assessment and data maintenance and storage. The project manager (ARACY) and research manager (CCCH) will be responsible for creating and maintaining study documentation.

Figure 1: right@home governance structure

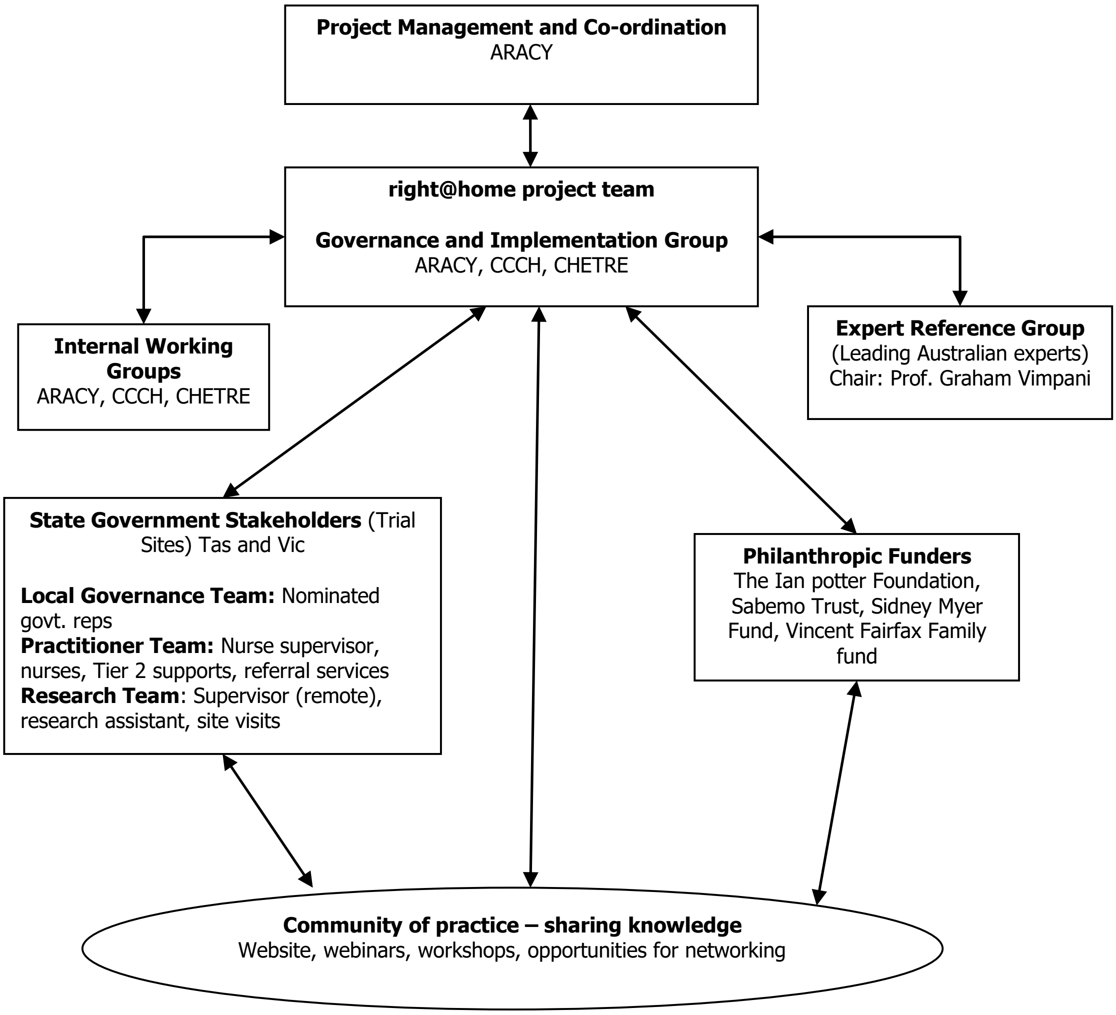

1.2.1 Principal Investigators

A/Prof Sharon Goldfeld,
The Centre for Community Child Health
The Royal Children's Hospital
Flemington Road
Parkville, VIC 3052
sharon.goldfled@rch.org.au

T: +61 3 9345 6408
F: +61 3 9345 5900

Dr Anna Price
The Centre for Community Child Health
The Royal Children's Hospital
Flemington Road
Parkville, VIC 3052
anna.price@mcri.edu.au
T: +61 3 9345 6355
F: +61 3 9345 5900

1.2.2 Statistician

Dr. Fiona Mensah
The Centre for Community Child Health
The Royal Children's Hospital
Flemington Road
Parkville, VIC 3052
fiona.mensah@mcri.edu.au
T: +61 3 9345 4741
F: +61 3 9345 5900

1.2.3 Internal Trial Committees

1. Expert Reference Group:

The Expert Reference Group (ERG) comprises 18 Australian health professionals with significant knowledge, practical experience and/or influence in the field of child health and wellbeing, maternal health and family health. They bring unique knowledge and skills to complement the knowledge and skills of the three partner organisations responsible for running the trial (CCCH, ARACY and CHETRE) to enable these partners to make informed decisions on some of the complex issues presented to them in developing the Sustained Nurse Home Visiting Model. The ERG also includes six government representatives from states participating in the trial, who represent the government and policy agenda in the trial.

A terms of reference document (see **Appendix 1**), outlines the role and expectations of ERG members and the governance and administration of the ERG. ERG members are:

- Prof Graham Vimpani (Chair), University of Newcastle, NSW
- Prof Kerrie Bowering, Women's and Children's Health Network, SA
- Mr Warren Cann, Parenting Research Centre, VIC
- Ms Karene Fairbairn*, Department of Education and early Childhood Development (DEECD), VIC
- Prof Sharon Dawe, Griffith University, QLD
- Mr Brendon Douglas, JTA International, QLD

- Prof Catherine Fowler, University of Technology, Sydney, NSW
- A/Prof Jennifer Fraser, The University of Sydney, NSW
- A/Prof Sharon Goldfeld, Centre for Community Child Health (CCCH), Murdoch Childrens Research institute (MCRI), VIC
- A/Prof Des Graham, Department of Health and Human Services (DHHS) – Children, Tasmanian Government, TAS
- A/Prof Harriet Hiscock, Hiscock CCCH, MCRI
- A/Prof Lynn Kemp, Centre for Health Equity Research and Evaluation (CHETRE), University of New South Wales (UNSW), NSW
- Prof Sue Kruske, Queensland Centre for Mothers & Babies, University of Queensland, QLD
- Ms Deborah Leisser*, DHHS – Children, Tasmanian Government, TAS
- Ms Christine Long*, DHHS – Children, Tasmanian Government, TAS
- Prof John Lynch, University of Adelaide, SA
- Dr Tim Moore, CCCH, MCRI, VIC
- Prof Victor Nossar, Department of Health and Families, NT
- Dr Anna Price, CCCH, MCRI, VIC
- Mr Luke Hatton*, DEECD, Vic (took over from Mr Anthony Raitman in 2013)
- Prof Michael Sawyer, University of Adelaide, SA
- A/Prof Virginia Schmeid, University of Western Sydney, NSW
- Ms Mailin Suchtin*, Department of Health and Ageing, NSW
- Prof Cate Taylor, University of Western Australia, WA
- Mr Mike Willie*, DHHS – Children, Tasmanian Government, TAS

There have been two face-to-face ERG meetings and seven teleconferences held in the first year of the sustained nurse home visiting (SNHV) trial (2012). It is anticipated that this group will meet via teleconference on a bimonthly basis over the next three years. In addition, there is regular email correspondence between group members and the partner organisations.

2. Government Stakeholder Group:

The six representatives from the state governments (denoted with asterisk (*) above) involved in the SNHV trial participate in ERG activities described above. In addition, the partner organisations conduct fortnightly meetings with a representative of each stakeholder group and conduct 6-weekly face-to-face project update meetings with both the Tasmanian and Victorian governments.

1.3 Sponsor

Murdoch Childrens Research Institute

1.4 Funding and resources

This overall study is financed by a combination of monetary and ‘in-kind’ support from the following institutions and departments. The research component of this study is funded by the four philanthropic bodies and these funds are provided to MCRI from ARACY through a standard contractual arrangement:

- Victorian DEECD
- Tasmanian DHHS
- The Ian Potter Foundation
- Sabemo Trust
- Sidney Myer Fund
- Vincent Fairfax Family fund

The current funding supports a four-year trial, i.e. follow-up of families to child age 2 years. Given that the overarching aim of the trial is to improve children's development and early learning, ideally families would be followed-up until children reach school-entry age (at 3 years and again at 5 years), when these outcomes can be reliably measured. As such, the research team intends to apply for an NHMRC Partnership grant and ongoing government and philanthropic support to fund ongoing follow-up. We also intend to seek ethical approval (via future modifications) to seek consent to link data with existing datasets to obtain data at these later follow-ups, with minimal burden for the participating families, e.g. Medicare, Perinatal Health, Maternal and Child Health, Centrelink, School Entry Health Questionnaire, NAPLAN. The Baseline PIS&C (**Appendix 2**) includes some information about the future data linkage consent request.

2. INTRODUCTION AND BACKGROUND

Background Information

Research has demonstrated that children raised in vulnerable families, including those affected by economic or social disadvantage, are at particularly high risk of poorer cognitive, emotional and behavioural outcomes in later childhood[1].

Early intervention programs that seek to address these issues can alter the developmental trajectories of highly vulnerable children, while improving outcomes for parents. The evidence suggests that the most successful early intervention programs combine strategies that target both children and their parents[2]. Commonly used early intervention strategies are sustained nurse home visiting (SNHV), childcare, and parenting education[3].

A recent literature review of SNHV programs[4] (included in **Appendix 3**) completed by CCCH concluded that when vulnerable families with risk factors for adverse child outcomes are partnered with a qualified nurse, from pregnancy until the child is 2 years of age, child and parent outcomes can be improved over the medium- to long-term. The SNHV model is preventative and non-stigmatising and encourages partnership between the nurse and the family to promote child health and family functioning.

By promoting safe, stable and nurturing relationships and environments, SNHV interventions have strong potential to empower parents to become confident carers. Evidence points to lasting impacts on mothers, children, families and entire communities, including less child abuse and neglect, more

employment for the mothers, and better performance at school for their children[5]. Importantly, the approach is tailored to respond to the specific needs of vulnerable families, and is holistic. Most services focus on one or other member of the parent-child dyad; however, in the SNHV program we will deliver an intensive and responsive service that meets the needs of both the family and child.

While there has been substantial international literature to support nurse home visiting,[4] there has only been one modest randomised controlled trial of this approach in Australia. Kemp and colleagues from CHETRE conducted the Miller Early Childhood Sustained Home Visiting (MECSH)[6] program in a single, socioeconomically-disadvantaged suburb in greater Sydney. The authors aimed to improve family, maternal and child health and developmental outcomes. At 2 years of age, mothers offered the MECSH program provided a more supportive home environment for their children, e.g. were more responsive, provided appropriate play materials, were more involved with their child, and the environment was better organised, than control mothers.[7] Intervention mothers also reported an improved experience of being a mother, and those born overseas breastfed for longer. There were no differences between groups in parent-child interaction and child development, maternal health and family, or immunisation and smoking outcomes.[7] The results from this relatively small trial suggest that further trialling in a larger sample would provide the evidence necessary to implement a program of this scale and intensity across Australia.

Given the potential benefit of such a program, it is opportune and necessary to undertake such a trial to measure benefit and cost effectiveness in the current Australian service system and policy environment. The new SNHV trial is being coordinated by the three partners: CCCH, ARACY and CHETRE. By building on and further developing the MECSH intervention, the aim of the main right@home RCT is to investigate whether a SHNV program, offered to vulnerable Australian mothers from the antenatal period to child age 2 years, improves parental care and responsivity with the child, and a supportive home learning environment at child age 2 years, with the ultimate goals of improving children's early learning and development by the time of school-entry. The main right@home trial has received substantial financial investment from the Victorian and Tasmanian state governments and philanthropy to run across 7 trial sites in the two states (4 in Victoria, 3 in Tasmania).

2.1 Research Question

Does a sustained, nurse home visiting program, offered to vulnerable mothers in Australia from the antenatal period to child age 2 years, improve parent care and responsivity, and the home learning environment at child age 2 years?

2.2 Rationale for Current Study

This study is crucial for generating Australian evidence of effective interventions to reduce the impact of social and environmental factors predisposing infants and children to ill health and reducing their life potential, an issue of timely and significant policy interest at a state and federal level[8]. Critically, the rigour and scope of this trial will determine the impact of a comprehensive Australian SNHV program commencing antenatally in an at-risk population group. Despite the rhetoric regarding the benefit of SNHV, this will be the first trial in Australia to test its benefit and cost-benefit both at scale and within the context of a population health conceptual framework (i.e.

not within a child protection framework). As such, this trial is a best-practice implementation and effectiveness model for professional home visiting in Australia, with significant implications for the development of early childhood policy and strategy throughout Australia.

3. STUDY OBJECTIVES

3.1 Primary Objectives

The overarching aim of the right@home trial is to improve children's development and early learning so that school readiness and subsequent academic performance is improved. Currently we are only funded to follow children to age 2 years. Our team's thorough review of the measures available at 2 years indicates that very few reliably predict children's development and learning at school-age. Instead, we argue that assessing the parent and environmental factors that underpin healthy development and learning will provide more measurable and hence meaningful outcomes for the study. Based on discussions making use of the ERG, the research team members' expertise and knowledge of this field, and three reviews of the literature (e.g. Appendices 3 and 30), the team have chosen primary outcomes that support the brain areas involved in early development and learning (areas thought to be particularly susceptible to the environmental factors associated with disadvantage)[9] and parenting outcomes. Thus, the primary objectives of this study are to evaluate, at child age 2 years old, the impact of a SNHV program offered to vulnerable Australian mothers, on:

1. Parent care: the parent's ability to provide a consistent, regular and supportive environment for their child-promoting child regulation
   - 1.1 Nutrition: e.g. consistent meal times, nutritious food
   - 1.2 Sleep: bedtimes, settling and infant crying
   - 1.3 Safety: internal and external home safety
2. Parent responsivity: the parent's ability to tune in to their child's needs and to respond appropriately; and
3. Home environment: building a strong home learning environment through structured developmental promotion activities focusing on language.

The measures for these outcomes are outlined in Sections 4 and 7.

3.2 Secondary Objectives

The secondary objectives of this study are to evaluate, across child age 6 weeks to 2 years, the impact of a SNHV program offered to vulnerable Australian mothers on:

1. Maternal: Pregnancy outcomes, Quality of Life, Mental health, Parent wellbeing, General Health, Parenting self-efficacy, and Health service use;
2. Child: General health and functioning;
3. Sibling: Mental health/behaviour; and
4. Nurse: Ability to effectively engage with parents and Use and knowledge of evidence-based clinical strategies.

4. STUDY DESIGN

4.1 Type of Study

This is a longitudinal, randomised controlled, multi-site evaluation of a sustained nurse home visiting program compared with usual care. The program will be offered to families from the antenatal period to the final follow-up assessment at child age 2 years. Researchers responsible for collecting the data, but not families or nurses, will be blinded to participating families' randomisation status.

A critical feature of the current trial is that it is designed based on strict program logic (see Figure 2 below), which is lacking in many of the previous home visiting randomised trials. A recent review by Segal et al (2012)[10] of home visiting programs to reduce child maltreatment showed that programs based on strict program logic were much more likely to be effective than those that were not. As such, we are designing the trial to ensure any effects can be clearly traced back to the intervention offered. Specifically, the right@home intervention is based on the MECSH SNHV program, but is being enhanced to include evidence-based strategies on three focus modules which specifically target the three primary outcomes measured at age 2 years: parent care and responsivity, and a supportive home learning environment.

Figure 2: Program logic for the right@home trial

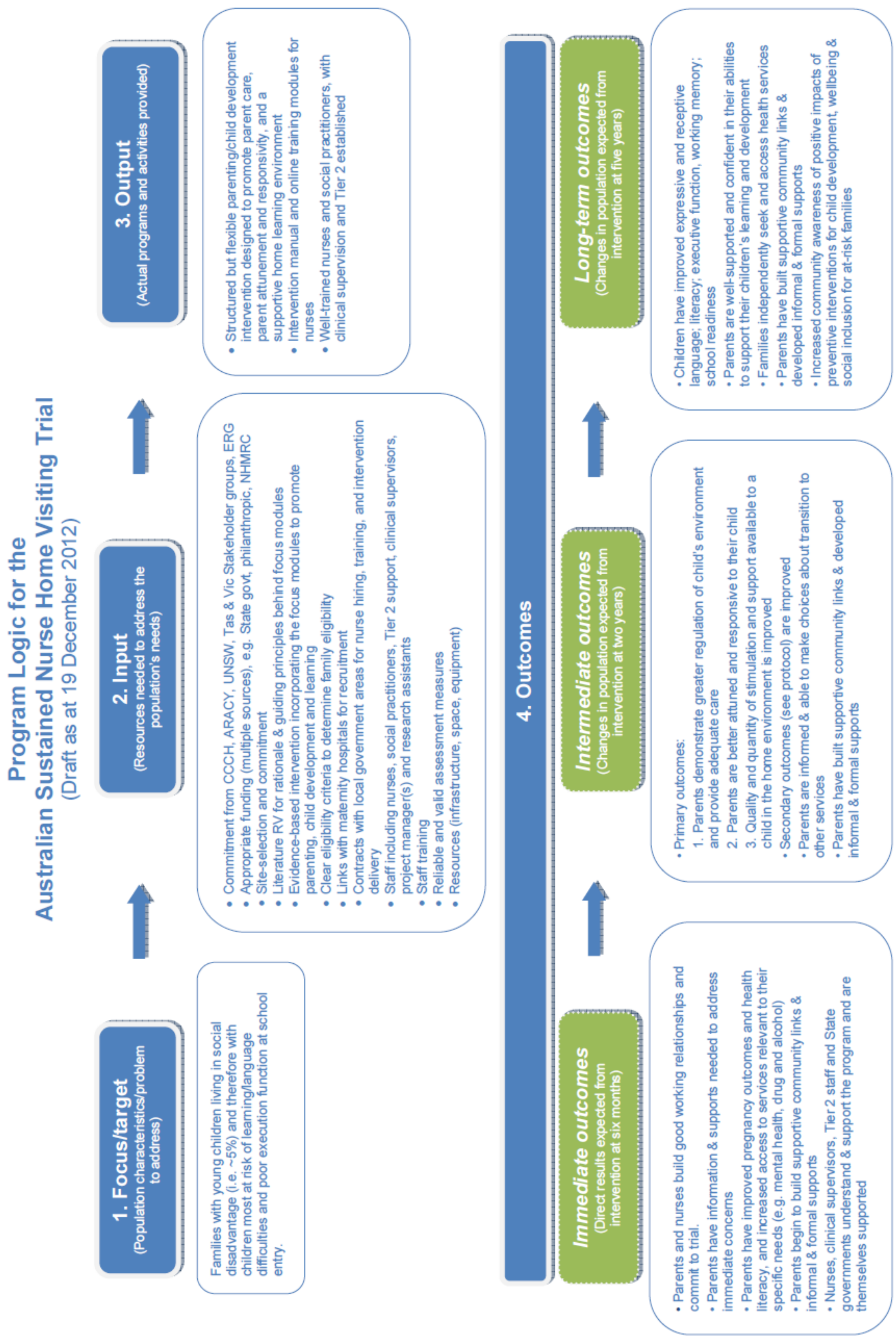

4.2 Study Design Diagram

Figure 3: Study Design Diagram

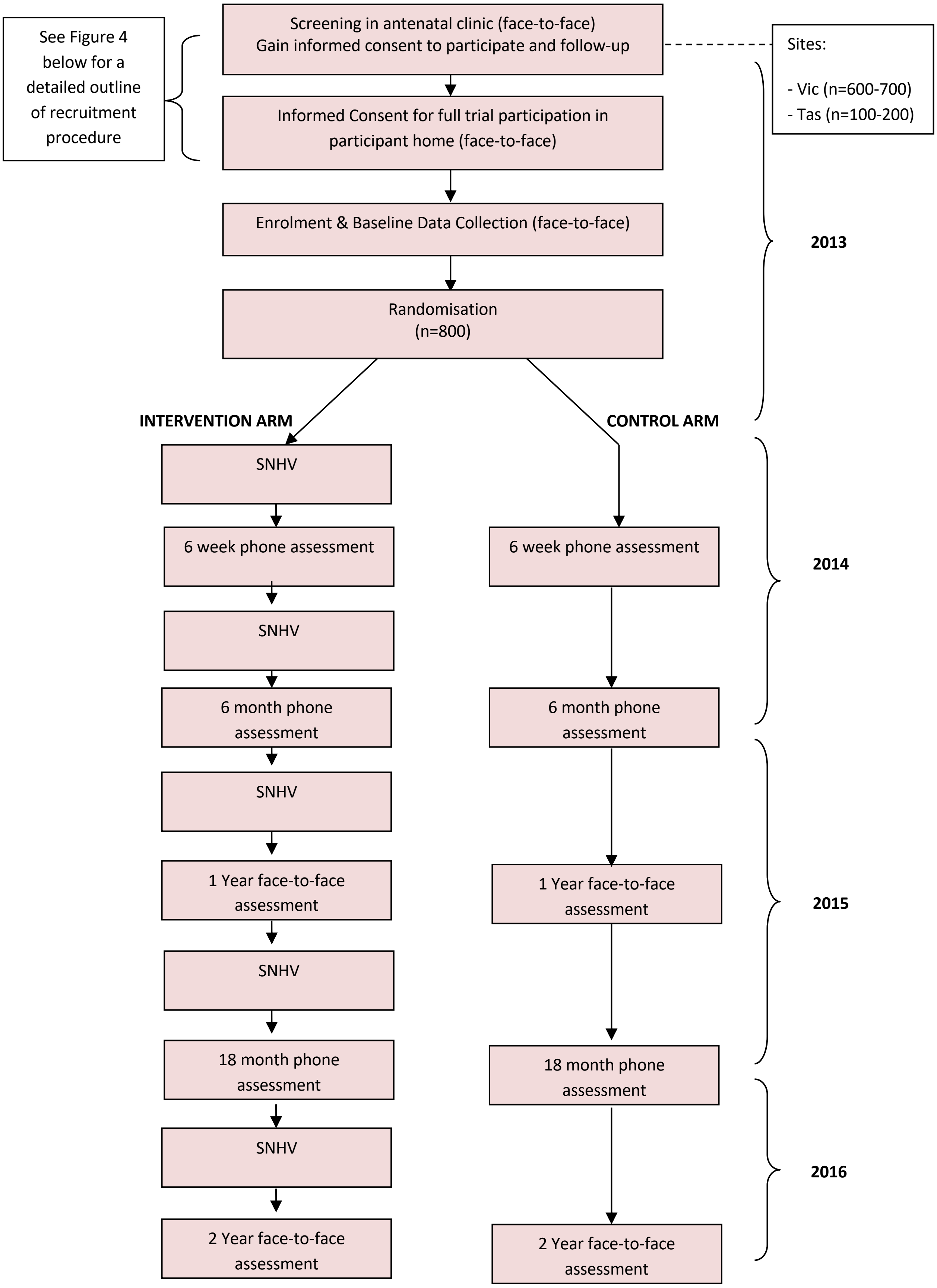

Figure 4: Detailed Study Design Diagram for Recruitment

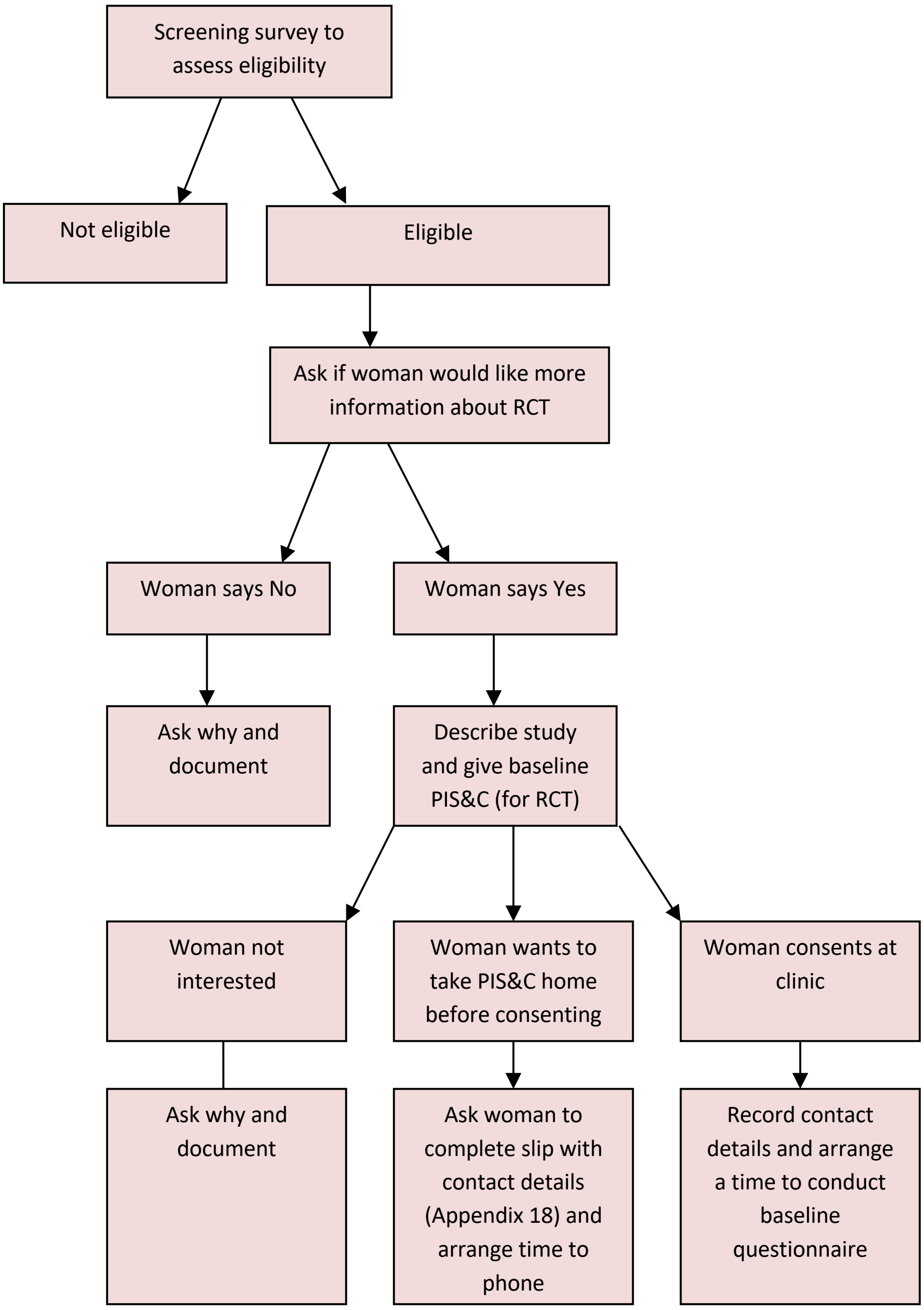

Number of Subjects

An anticipated n=800 pregnant vulnerable mothers (see Eligibility criteria, Section 6.2) will be enrolled into this study (n=400 per arm).

4.3 Expected Duration of Study

The expected duration of the study is 5 years. Recruitment will take place over 12 months (2013) and families will be offered the intervention and followed for 2.5 years (2014-16). Final analysis, publication and reporting will take place in 2016-17.

4.4 Primary and Secondary Outcome Measures

Primary and secondary outcome measures are listed in Table 2 below, and the schedule of these measures is presented in Section 7. A description of the baseline measures and their psychometric properties is attached in **Appendix 4**. Measures have been chosen for:

- Meeting the study's strict program logic, i.e. as direct measures of the three primary areas of intervention,
- Acceptability to women and the feasibility of collection relative to cost and time burdens, and
- Where possible, strong psychometric properties, having normative data available for population comparisons and previous use in international and/or Australian published research, particularly home visiting trials.

As there are three targeted areas of intervention, there are three **primary outcomes**: (1) Parent care, (2) Parent responsivity, and (3) Supportive Home Environment.

Table 2: Primary and Secondary Outcome Measures

Measure	Description
Demographics	E.g. psychosocial and socioeconomic risk factors collected via self report
Contact details	Family and alternate contact details
PRIMARY OUTCOMES	
1. Parent care:	
Nutrition: child height and weight, mealtime diary report	Child height and weight collected via direct observation; study-designed diary based on LSAC diary measuring types of food consumed in 24-hours, collected via parent report.
Safety: Home safety audit	Observational safety check to identify household hazards, adapted from the Safe Place Project intervention.
Sleep: Sleep problems	Single parent report item drawn from LSAC: "How much of a problem is your child's sleep", responses "none", "mild", "moderate", and "large".

**Measure**	**Description**
2. Parent responsivity:	
Parent responsivity subscale (observational) of the Home Observation for Measurement of the Environment Inventory (HOME)	Quality and quantity of stimulation and support available to a child in the home environment. Assessed through observation and interview with the primary caregiver (usually the mother) of the child in the family home.
3. Home Environment:	
Confusion, Hubbub and Order scale (CHAOS):	Parent report scale specially designed to be administered to parents for assessing chaos in the child's home. A measure of confusion and disorganisation.
HOME	See above (observational and parent report subscales)
MacArthur Communicative Development Inventory (MCDI)	Standardised parent report questionnaire to assess children's receptive and expressive language.
SECONDARY OUTCOMES	
Maternal	
Pregnancy Outcomes	E.g. self-reported length of breastfeeding, immunisations
Family Psychosocial Screening Instrument	Parent report scale that assesses domestic violence, parental substance abuse, and social support/capital.
Assessment Quality of Life (AQoL-8)	Self-reported multi-attribute utility health-related quality of life instruments for use as utility instruments which measure quality of life.
Maternal mental health: Depression, Anxiety, Stress Scale (DASS-21)	Three self report scales designed to measure the negative emotional states of depression, anxiety and stress.
Parenting Wellbeing Index (PWI)	Self report questions about life satisfaction with 8 specific life domains. The domains are theoretically embedded, as representing the first level deconstruction of the global question: 'How satisfied are you with your life as a whole?'
General Health Questionnaire (GHQ-S1)	Single self report item assessing overall health
Parenting self-efficacy	Self report items drawn from the Millennium Cohort Study, assessing how the mother feels about her life so far, e.g. the extent to which she feels that she gets what she wants out of life, feels in control and can run her own life.
Health service use	Self report questions designed for the study in consultation with investigators Gold and Flego (right@home health economists)
Health literacy	Study-designed self report items assessing mothers' knowledge, attitudes and beliefs about paediatric health
Parenting styles	Three self report subscales drawn from LSAC, assessing parenting warmth, hostile parenting and inconsistent/irritable parenting.

Measure	Description
Child	
General functioning: Pediatric Quality of Life Inventory (Peds-QL) – Infant Scales	Parent report scale that assesses a child on 5 subscales (physical functioning, physical symptoms, and emotional, social and cognitive functioning).
General Health: GHQ-SF1	See above
*Sibling**	
Mental health/behaviour: Strengths and Difficulties Questionnaire (SDQ)	Parent report scale designed to measure psychological adjustment in children (collected for right@home siblings aged 3-6 years old only).
*Nurse***	
Dose, fidelity and clinical knowledge/practice	Study-designed participant report items assessing use and knowledge of evidence-based clinical strategies

* The SDQ will be collected (where parents agree) for all siblings aged 3-6 years as 3 years is the earliest age that the SDQ can be administered, and it is unlikely that the right@home intervention will have secondary effects on school-age children.

**The dose of the intervention delivered and nurse fidelity will be measured via data collected after each home visit a nurse conducts.

5. STUDY TREATMENTS

5.1 Treatment Arms

5.1.1 Description

Service design concept: Right@home has been designed as a proportionate[11] (i.e. higher dose and intensity) service response from the universal maternal and child health services in Victoria and Tasmania. As such, the intervention embeds the universal aspects of the existing service system. In Victoria there are ten Key Ages and Stages consultations recommended to review a child's health, growth and development from birth to 3 ½ years. These include: a home visit and consultations at 2, 4 and 8 weeks, 4, 8, 12 and 18 months, 2 and 3 ½ years of age for all children and their families.

In Tasmania the local Child and Family Health Nurses offer child health and development checks at similar time points, the only difference is that a 6 weeks medical check is recommend and there is no check between 18 months and 3 ½ years.

Intervention group: The MECSH program (see detailed manual in **Appendix 5**) is the foundation for the right@home program. Built onto the MECSH program are a number of evidence-based strategies that are intended to directly impact the three primary outcomes. These evidence-based strategies, termed 'focus modules', were identified in another recent CCCH literature review[12], informed by the ERG and reflect best national and international practice. They include strategies on infant sleep training; nutrition; safety; regulation; attachment and social support; and video feedback and

motivational interviewing techniques. The extra modules are currently being incorporated into the right@home program and will be finalised by early March 2013 (see draft addendum to manual in Appendix 6, attached in confidence).

The intervention group will receive at least 25 home visits (actual number of visits determined by need) primarily by the same SNHV program nurse during the remainder of pregnancy and the first 2 years post birth. The nurse will be provided with clinical supervision and each group of nurses (up to 4) will be supported by a dedicated social care practitioner for them and their families. Staff and resources for the intervention will be funded by the industry partners and managed by ARACY and CHETRE. The SNHV nurses are MCH nurses who have received additional training in the intervention program, operating within a distinct SNHV team. Each nurse will be expected to have Family Partnership Training (see http://www.fpta.org.au/). The first six right@home nurse training modules are described in **Appendix 7** (attached in confidence).

The home visits are standardised as follows:

a) Antenatal and postnatal care in accordance with the MCH and MESCH guidelines: antenatal home visits at least 2nd weekly and postnatal visits within one week of birth, and then at least weekly until 6 weeks; second weekly till 12 weeks; monthly to 6 month; bi-monthly until 2 years.

   The content of home visits will predominantly focus on evidence-based strategies underpinning the three primary outcomes: the care of child, responsivity to child, and providing a supportive home environment.

   The content of each home visit will be individually tailored to each mother's needs, skills, strengths and capacity. Guided by a strengths-based approach and goal setting, the nurse will support and enable the mother and the family to:

   - enhance their coping skills, problem solving skills and ability to mobilise resources;
   - foster positive parenting skills;
   - support the family to establish supportive relationships in their community;
   - mentor maternal-infant bonding and attachment; and
   - provide primary health care and health education, including but not limited to evidence-based information regarding immunisation, Sudden Infant Death Syndrome (SIDS) risk reduction, infant nutrition and child safety.

b) Postnatal Learning to Communicate[7] program consisting of 12 monthly sessions commencing when the baby is one month old and finishing when the baby is 12 months old, which include information and activities for parents to encourage child development. Sessions 1-5 are delivered individually during the home visits. Sessions 6-12 are delivered in a group environment. The effectiveness of the Learning to Communicate program has been demonstrated in an unpublished controlled trial, and is a published clinical resource used by clinicians throughout Australasia.

c) Access to early childhood health services, volunteer home visiting services and family support services.

d) Group activities and community links including parenting group and walking group specifically for intervention families, and linking into community activities.

The intervention incorporates the standard Key Age and Stage (KAS) visits described in the Service Design Concept above. Existing MECSH nurse visit checklists (see examples in Manual in **Appendix 5**) will be modified to include statements regarding the need to do KAS at the appropriate times.

Usual care (control) group: The control group receive the usual care; that is:

a) Antenatal care according to MCH protocols, which include:

- One postnatal home visit by a nurse from the regular child and family nursing service (within 2 weeks of baby's birth)
- Additional postnatal home or clinic visits with the regular child and family nursing service as indicated by protocols in usual care.

b) Volunteer home visiting services and family support services within the postcode area as available

Being in the right@home study (both control & intervention) will not preclude access to other support services, except for being part of the Cradle to Kinder (CtK) programs (see Exclusion Criteria, Section 6.2).

Hence the key differences between the right@home intervention and usual care are:

- Home visiting commencing antenatally;
- Continuity of care by the same nurse throughout the 2½ year program;
- Care by nurses with additional training in the right@home and MECSH program model;
- Standardised postnatal home visiting program to the child's second birthday;
- More frequent, sustained and responsive visits in right@home
- Dedicated social care practitioner;
- Learning to Communicate program and specialised toolkit of focus modules;
- Group activities and proactive links to community activities.

5.1.2 Dosage and Route of Administration

The intervention will be delivered face-to-face by the MCH nurse to the participating woman and any family members she chooses to be present. Nurses will deliver a minimum of 25 visits over the

course of the intervention. Nurses are recommended to limit the total number of visits to 35. It is suggested that families with a greater need should be referred to other community supports and health providers for additional support.

5.2 Measurement of subject compliance

Subject compliance will be measured with fidelity instruments completed at every home visit, which are yet to be finalised but will be based on those used for MESCH (see **Appendix 8** and **Appendix 9** for antenatal and postnatal checklists, respectively). The checklists are used to record intervention delivery and fidelity only, and will not be seen by researchers assessing families. We will submit the fidelity measures for ethical approval once finalised for right@home. Significant non-compliance is defined as missing 6 months of the program, i.e. if no attendance for 6 months.

5.3 Excluded medications and treatments

Families taking part in the Victorian Department of Human Service Cradle to Kinder (CtK) program or the Tasmanian CU@home program will not be eligible for the right@home study. CtK is an intensive support program for very vulnerable, teenage Australian mothers to prevent involvement in the Child Protection System. CU@home is a home visiting program designed for first-time mothers aged 15-19 years old. CtK and CU@home families are excluded from right@home because the programs are too intensive and complex for families to participate in both. The total number of CtK families is small (i.e. n~250) and recruited across Victoria, so we expect very few CtK families to be eligible families from the three Victorian right@home sites. Similarly, Tasmanian first-time mothers aged 15-19 years will only make up a small proportion of the right@home sample, and only around half of these mothers engage with the CU@home program.

6. SUBJECT ENROLLMENT AND RANDOMISATION

6.1 Recruitment

Pre-recruitment preparation: Following introductory emails sent by Maya Rivis (Victorian Department of Health) to Victorian antenatal clinic managers, and Christine Long (Tas Department of Health and Human Services) to Tasmanian antenatal clinic managers (see **Appendix 10** and **Appendix 11**), our research team has been in contact with managers at each antenatal hospital to discuss the right@home trial and identify which locations the right@home recruitment could feasibly take place. The local councils listed in Section 1.1 have signed or are in the process of signing contract agreements (see Appendices 25-29 for completed contracts). Since August 2012, our research team has had ongoing email and phone contact with the maternity staff at each site to determine the specifics of recruitment, e.g. to ascertain birth rates, to decide which clinics our research staff will recruit from, and to answer questions raised by the antenatal managers.

At the beginning of each recruitment session, i.e. before an antenatal clinic begins, researchers will briefly talk with clinic staff and provide a one-page information sheet that describes the study to staff (**Appendix 12**). Many of the clinic staff will already know about the study, but this ensures that all clinic staff know about right@home in a systematic and standardised way. An A3-sized poster describing the right@home study (**Appendix 13**) will be displayed in each antenatal clinic and, if women request information about the study before the researcher has an opportunity to speak with them, clinic staff can refer women to a flyer that our research team will print and make available in each waiting room (**Appendix 14**).

Recruitment: Potential participants will be all women who are pregnant, attending the antenatal clinics at the site hospitals listed in Section 1.1 and who fulfil the eligibility criteria outlined in Section 6.2. The recruitment and assessment process for the right@home trial are summarised in the Study Design Diagrams in Section 4.2. A short screening survey based on the pilot survey (see **Appendix 15**) will be used to identify eligible women for the right@home trial (see Eligibility criteria in Section 6.2). A trained research assistant will attend each antenatal clinic and approach all women in the waiting rooms. The researcher will describe the study (see script in **Appendix 16**) and ask whether women might be interested in taking part. At this first contact, researchers will ask women to reiterate what they are asking of the potential participant (see **Appendix 16**), to ensure the woman has understood the request. Women who do not demonstrate comprehension (e.g. require an interpreter for insufficient English or have intellectual disability) will be excluded from the trial.

This initial survey will take place in the hospital waiting room as not all hospitals have access to private rooms. Every effort will be made to ensure all conversations take place as discreetly as possible and are sensitive to the surrounds. Women who express interest in the study will be asked to provide signed, informed consent (see **Appendix 17**) before completing the screening questions. In our pilot of 118 women there were no concerns expressed by women in either being approached or completing the survey in the waiting room. This first consent process indicates that the woman is happy to participate in the screening survey, and to hear more about the RCT if eligible. If the screening survey shows that a woman is eligible for the RCT, the researcher will tell her this straight away (see script in **Appendix 16**) and ask her if she would like to hear more about the trial. Women who say yes will be told more about the study (see script in **Appendix 16**), given the baseline PIS&C and asked if they would like to read through the PIS&C then, or take it home. Women who provide informed baseline consent at that point will be asked for their contact details and asked if they would like to make a time for the first home visit then. Women who want to take the PIS&C home to read first will be asked if they are happy for the research team to phone them in a few days to follow-up. Women who say yes will be asked to complete a contact details slip (**Appendix 18**). Women who are not interested in hearing more, or who read the PIS&C and then decline participation, will be asked for a reason why (if they are happy to give a reason). Before a woman leaves the clinic, the researcher will give her a card (**Appendix 19**) with the study team's contact details, and encourage the woman to contact the team if they have any further questions about the study.

In the days following the antenatal screening survey, researchers will phone women who provided their contact details to answer any questions. Researchers will also ask whether the woman is part of the Victorian CtK program. Women who are part of the program will be informed that they are

ineligible for the right@home study, and will cease participation. Researchers will organise a suitable time for the first home visit with women who provide informed consent for the RCT (baseline PIS&C) at the antenatal clinic. Women who provided contact details but not informed consent will be asked if they are happy to take part and, for those who agree, the researchers will organise a time for the first home visit. For women who decline participation, researchers will record reasons why, if the woman is happy to specify.

Enrolment: At the enrolment home visit with the researcher, a signed consent form will be obtained for each participant before any further survey administration. Women younger than 18 years are not excluded from the study because they are part of the vulnerable population that this trial targets. These women will be making their own decisions about participation in health services for themselves and their child. Some pregnant young people are estranged from family and it would be inappropriate to require parental consent in these circumstances. In line with Section 4.2.9 of the National Statement, we will assess their understanding of the information by verbally going through the information sheet (this will be standard practice in this research as levels of English-language literacy will not be assumed), and then asking the young person (and indeed all participants) to reiterate to the researcher what they understand the research to involve and whether they needed further clarification. We believe this is best practice for this population and enables appropriate discussion of the consent process. In this way we would take steps to ensure participants understand the relevant information. . Furthermore, with regards to the rest of section 4.2.9, this research is low risk, is very likely to benefit the young person, and may be contrary to the best interests of the young person to seek consent from parents. Where the young woman would like to do so, we will also explain the research to, and gain consent from their parents.

The consent form will describe the purpose of the study, the procedures to be followed, and the risks and benefits of participation. The trained researcher will conduct the informed consent discussion and will check that the participant and their legally acceptable representative comprehend the information provided and answer any questions about the study. Consent will be voluntary and free from coercion. At all times it will be made clear that non-participation in the study will not affect the usual routine clinical management offered by any health providers, e.g. the care they receive from the hospital or as part of the maternal and child health service. The researcher who conducted the consent discussion will also sign the informed consent form. A copy of the consent form will be given to the participant or their legally acceptable representative and the fact that the participant has been consented to the study will be documented in the participant's record on the study's electronic database.

Following the baseline assessment and once all the inclusion and exclusion criteria have been addressed and the eligibility of the participant confirmed, allocation to the intervention or usual care arms will be conducted online by the research assistant using a web-based randomisation application set up by an independent statistician. We aim to recruit 20 participants (10 per arm) per week until the total number of participants is obtained. The research assistant will then inform the participant of their randomisation status.

6.2 Eligibility Criteria

There have been a number of considerations in establishing the eligibility criteria and thus the recruitment strategy for the right@home trial. These include:

1. Longitudinal research about antenatal predictors of maternal and child outcomes. One of the key finding's from CCCH's first literature review[4] (**Appendix 3**) was that home visiting programs are more effective for families of low socioeconomic backgrounds. However, SNHV trials typically recruit based on a small number of risk factors (e.g. teenage parent, poverty). As such, it is not possible to say whether women with other known risk factors for poorer child development and learning outcomes may benefit from SNHV programs. For example, Chittleborough et al (2012)[13] found that antenatal maternal factors such as teenage parent, single parent, low education, perinatal depression, poverty and smoking were strongest predictors of poor parent-infant related outcomes. PREview (http://www.chimat.org.uk/preview/evidence) indicates that postnatal (child 9 months) maternal predictors of child health, learning and behavioural outcomes at age 5 years were low income, low education, poor general health and low self efficacy. Following from this, we selected the risk factors to be tested by the recruitment pilot to encompass a range of socioeconomic and psychosocial characteristics.

2. Important "success" factors in previous SHNV trials relate to cohort "vulnerability". Summaries of the SNHV literature point to the added value of these programs for more vulnerable families, particularly those with low psychological resources and supports. Research reviews have also recognised that, while the most vulnerable might be those most likely to benefit, they are also hardest to recruit and retain in studies. Risk factors cluster together and, for the purposes of the right@home trial, we are assuming that the greater number of risk factors a woman has, the more 'vulnerable' she is.

3. Risk factors of the MECSH population. These included maternal age under 19 years, EPDS score ≥10, lack of emotional and practical support, late antenatal care, major stressors in the past 12 months, current substance misuse, current/history of mental health, history of abuse in mother's childhood and domestic violence. As the right@home trial builds off MECSH, we incorporated these risk factors into the pilot screening survey, to assess whether they were useful markers of vulnerability.

4. Pilot study to inform right@home eligibility criteria and recruitment procedure.

Background

For the right@home pilot recruitment study, we hypothesised that a screening survey delivered in antenatal waiting rooms – with questions targeted at maternal risk factors, particularly those known to be important for future child health – would capture vulnerable women likely to benefit from the right@home SNHV intervention. The screening survey items included age (calculated from year of birth), relationship status, social support, general health, having a long-standing illness, anxious mood, education level, current employment, and proxy measures for poverty (whether the woman receives Centrelink benefits and/or owns a healthcare card (HCC)).

To test this hypothesis, we linked the screening survey data with midwifery-collected data recorded on the hospital's electronic Birthing Outcome System (BOS), which includes more sensitive risk factors (i.e. those that can be more difficult for women to feel comfortable disclosing in a public waiting room). These variables included smoking during pregnancy, alcohol use, drug use, marijuana use, social issues, domestic violence, a history of mental health problems, and an EPDS score.

Results

Sample characteristics: The 3-day pilot study was conducted at Northern Hospital, Epping. Of 118 women approached, 102 (86.4%) took part. The survey data were near-complete (few missing data) and, when asked, women did not express concern with the survey questions, suggesting that the survey was acceptable to women and feasible to collect in busy antenatal clinic waiting rooms. Women ranged in age from 18-41 years. The length of women's pregnancies ranged from 0-40 weeks, skewed towards the later months (median 32 weeks; interquartile range 24, 35 weeks).

81/102 women (79%) consented to having their screening survey data linked with their midwife-collected BOS data. The number and proportion of women with each risk factor is listed in Table 3 below. We excluded the Centrelink, HCC, and current employment risk factors from the agreement analysis (described below) because they did not differentiate risk in the sample, i.e. large proportions of the sample responded positively to the items. After these, the most common waiting room risk factors were young pregnancy (<23 years), poor/fair/good general health, not finishing high school and no previous employment.

Agreement between screening survey and BOS data: Of the 81 women with complete data, 21 had any 1 or more BOS risk factors (proportions also presented in Table 3 below). Of these, 6 were current smokers only with no other BOS risk factors, and 3 were current smokers with at least one other BOS risk factor. Six women had BOS risk factors but no waiting room risk factors and, of these, four had the single BOS smoking risk factor and no others. This means that if we add smoking status to the waiting room screening survey items, we would only miss only two women with BOS risk factors (2/21, 9.5%) of the participating waiting room population.

Our research team, including CI Mensah (statistician), examined different algorithms for identifying which eligibility criteria would identify the most vulnerable women. These analyses demonstrated that choosing women based on 2 or more of the following 10 screening survey risk factors would sufficiently identify vulnerability: current smoking, young pregnancy (<23 years old), no support during pregnancy, poor/fair/good general health, anxious mood, not finishing high school, not having a household income, long-term illness, living without another adult, and not working previously. For example, of the 21 with a BOS risk factor, 12 (57%) were captured with 2 or more of the above risk factors. Of the 81 participants with complete data, 24 were captured (30%) and, of the total sample screened, 32/102 (31%) were captured.

We therefore intend to use these ten risk factors to screen women for the main right@home trial. However, an overall eligibility rate of 30% is high and may mean that some eligible women are not sufficiently vulnerable to benefit from the SNHV program. To identify these women, we will re-assess their vulnerability based on the comprehensive demographic information we collect at the baseline assessment, for the first 20 women enrolled. This will help us refine our eligibility criteria if necessary. In addition, as less-vulnerable women tend to self-select out of intensive parenting

programs (e.g. as in the MESCH trial), women and their nurses will be asked if they think they need the program. If both the woman and her nurse do not think the woman needs the program, the team will consider excluding the woman and refining the eligibility criteria. This will help prevent excessive attrition and protect study power.

Table 3: Proportions of participants with screening survey and BOS risk factors

	Sample, n (%)	
**Risk Factor**	**Complete data N=81**	**≥ 1 BOS risk factor N=21**
Screening survey		
<23 years old	10 (12.4)	2 (9.5)
No support	4 (4.9)	2 (9.5)
Poor/fair/good health	24 (29.6)	10 (47.6)
Anxious mood	5 (6.7)	3 (14.3)
<Year 12 education	19 (23.5)	9 (42.9)
No household income	5 (6.7)	4 (19.1)
Long-term illness	3 (3.7)	0
Living without adult	1 (1.2)	1 (4.8)
No previous work	10 (12.4)	1 (4.8)
No current employment	52 (64.2)	16 (76.2)
Centrelink benefits	37 (45.7)	15 (71.4)
HCC	32 (39.5)	11 (52.4)
BOS items		
Current smoking	9 (11.1)	9 (42.9)
Alcohol use	3 (3.7)	3 (14.3)
Drug use	1 (1.2)	1 (4.8)
Marijuana use	2 (2.5)	2 (9.5)
Social issues	3 (3.7)	3 (14.3)
Domestic violence	1 (1.2)	1 (4.8)
History of mental health issues	8 (9.9)	8 (38.1)
EPDS >12	1 (2.1)	1 (8.3)

Participants will be assigned to a randomised study treatment only if they meet all of the inclusion criteria and none of the exclusion criteria.

6.2.1 Inclusion Criteria

Vulnerable, pregnant Australian women, who have sufficient English proficiency to answer questions face-to-face. The inclusion criteria, based on the results of our pilot (RCH HREC 32771, Northern Health HREC P 20/12), are any two of the following 10 risk factors:

- Current smoking,
- Young pregnancy (<23 years old),
- No support (emotional, financial, practical) during pregnancy,
- Poor/fair/good general health (versus very good/excellent general health)
- Anxious mood,
- Not finishing high school,
- Not having a household income,
- A long-term illness,
- Living without another adult, and
- Never had a job.

6.2.2 Exclusion Criteria

Women will be excluded if:

- Their child is removed from the home during the course of the research such that they are ineligible for the trial. If it emerges that child is being removed from his/her home (i.e. intervention nurses inform the study team, or a researcher phone call to the mother to schedule an assessment reveals it), then the CIs Goldfeld and Price will consider whether the family can remain in the trial on a case-by-case basis. Remaining in the trial will depend on the length of time the child is removed from the home, as it is not possible to assess the effectiveness of an intervention if the family has no access to the intervention or usual care alternative. In some cases, it will only be possible to consider full exclusion of a family at the end of the trial.
- They are enrolled in the Victorian Department of Human Services 'Cradle to Kinder' research program
- They are enrolled in the Tasmanian Department of Health and Human Services CU@home home visiting program
- Do not comprehend the recruitment invitation (e.g. have an intellectual disability such that they are unable to consent to entering the study, or have insufficient English to complete face-to-face assessments, i.e. require an interpreter)
- Have no mechanism for contact (landline or mobile telephone, or email address)
- Experience a critical event such as miscarriage, late termination of pregnancy, stillbirth and neonatal death.

6.3 Randomisation Procedures

Patients will be randomly assigned to receive the treatment (home visiting intervention) or usual care arms. Families will be equally divided between the two arms, based on our sample size estimation (see Section 10.1). A statistician not directly involved in the analysis of the study results

will prepare the randomisation schedule using block randomisation to maintain balance between treatment arms. Randomisation will be stratified both by parity (1st time parent vs. those with children already) and site. The schedule will be administered using a web-based randomisation schedule to enable randomisation to be conducted remotely at each location. Sealed envelopes containing randomised treatment allocation will be provided to each study location in case of the event that the web based system cannot be accessed during the randomisation of a participant.

6.4 Blinding Arrangements

The project manager, participants and nurses will be aware of the allocation to treatment arm to enable organisation for the mothers to receive the active or control intervention. Research staff responsible for conducting outcome assessments will be blinded to treatment allocation. Families will be asked not to disclose their randomisation status at assessments; however, those that do will be recorded in the study database and this ‘unbinding’ will be examined as a potential confounding variable in the outcome analyses.

6.5 Breaking Of The Study Blind

6.5.1 On Study

Breaking of the study blind is not applicable as the study coordinator, mothers and nurses are aware of the allocation.

6.5.2 Following Completion of the Study

Research staff and the statistician responsible for the study analysis will be supplied with the treatment allocation for each participant when all mothers and children have completed the primary outcome assessment at 24 months.

6.6 Subject Withdrawal

6.6.1 Reasons for withdrawal

The research team may withdraw a participant from the study treatment and follow-up procedures if the participant:

- Is in violation of the protocol
- Experiences a serious or intolerable adverse event
- Develops, during the course of the study, symptoms or conditions listed in the exclusion criteria
- Requires early discontinuation for any reason
- Experiences a critical event such as miscarriage, late termination of pregnancy, stillbirth and neonatal death.

The latter will be identified via the research phone calls made throughout the study. We understand that it may be extremely difficult for women to share this information, so phone calls will be

conducted using the same sensitive procedure employed in other studies conducted at the MCRI. The researcher will begin the conversation by asking the mother how she is, which allows the mother to volunteer the information. For mothers in the intervention arm, intervention nurses are likely to learn of these events before the researcher. Intervention nurses will then pass this information back to the research CIs, and one of the CIs will contact the parent to express their sadness, take them off the database, and offer the maternal and child health service number in case parents want additional support. For control participants, the researcher conducting the phone call (CI Price or RO Bryson) will offer the same information, and ask whether the mother would like CI Goldfeld to contact their maternal and child health nurse to request more support.

At enrolment, women will be given study details on the PIS&C, and invited to contact the study team with any details they think we should know about. These processes have worked well in other MCRI studies.

The research team will withdraw all participants from the study treatment if the study is terminated. Patients are free to withdraw from the study at any time upon their request or the request of their legally acceptable representative.

6.6.2 Handling of withdrawals and losses to follow-up

If the participant is withdrawing from the intervention, the research team will ask whether they are still happy to take part in the research assessments in an effort to maintain the intention-to-treat analysis. If the participant withdraws fully from the study, they will cease to undergo scheduled assessments. Participants will be asked (only if willing) to share the reason for ceasing the intervention or participation in the full trial. Reasons will be recorded by the research team in the study database. Where it is not possible to contact a family, a limited number of follow-up processes will be conducted, including:

(1) 2 documented phone calls including alternative assessment settings offered,
(2) 2 documented emails (where email address are provided),
(3) Contacting Centrelink for updated contact details where consent to record the family's Centrelink is obtained. We are currently making inquiries into whether this is possible and will only be able to do it with the correct authority.

6.6.3 Replacements

Patients who discontinue the study will not be replaced by further recruitment to maintain the required sample size. Based on attrition data from previous home visiting randomised controlled trials, our sample size estimation (Section 10.1) has allowed for a potential attrition of 40% at 2 years.

6.7 Trial Closure

There are no circumstances under which the study will be terminated prematurely. The study may be extended if the recruitment rate is slower than anticipated and it is necessary to lengthen the recruitment period to reach the necessary sample size. In addition, we may extend the trial to follow-up the cohort until school age (5-6 years), to assess the effects of the home visiting

intervention on children's early learning and development. Extending the trial to assessments beyond age 2 years will depend on the success of future grant applications. We will seek participant consent for contact for these follow-ups and potential data linkage in future ethics modifications.

6.8 Continuation of Therapy

The home visiting intervention will not be offered to participants beyond the child's second birthday.

7. STUDY VISITS AND PROCEDURES SCHEDULE

The Study diagram in Section 4.2 and Figure 3 below depict the study visits schedule. From April 2013 to March 2014, researchers will approach women at antenatal clinics listed in Section 1.1 to invite women to take part in the screening survey (**Appendix 15**). At this point, consent involves agreeing to complete the screening survey and, if eligible, being invited into the right@home trial. Researchers will then visit interested and eligible women in their homes within 1-2 weeks of the initial screening procedure, to tell them more about the right@home study, and collect informed consent for participation in the randomised controlled trial and a thorough baseline assessment.

Once women complete the baseline assessment, they will be randomised into one of the intervention or usual care arms. Whether this randomisation takes place in the woman's home (at the end of the enrolment visit) or over the phone will depend on whether the intended electronic data collection processes can incorporate the web-based randomisation program, and whether the tablets can connect via 3G/wireless internet. Once a woman is randomised, her details will be sent to the right@home nurse coordinator responsible for that region, and the nurse will be responsible for initiating contact and beginning the intervention. Follow-up assessments will be conducted by the research team via phone at 6 weeks, 6 months and 18 months postpartum, and via face-to-face home visits at 12 and (the final follow-up at) 24 months.

Due to the anticipated low literacy level of the participant group, we are designing all assessments so that they can be completed face-to-face by interview. Responses will be recorded by researchers electronically on tablets, except for some sensitive questions (highlighted in the questionnaire), where the participant will be given the option to complete by themselves either by reading it or having it read to them.

Figure 5: Graphical depiction ('Perera diagram') of the components of the trial shared and unique to the intervention and control groups

Time point relative to randomisation	Intervention	Control
Screening (face-to-face)	A	
Informed consent (face-to-face)	B	
Enrolment & Baseline Data Collection (face-to-face)	C	
Randomisation		
SNHV	D	
6 week phone assessment	E	E
SNHV	D	
6 month phone assessment	F	F
SNHV	D	
1 year face-to-face assessment	G	G
SNHV	D	
18 month phone assessment	H	H
SNHV	D	
2 year face-to-face assessment	I	I

A	Screening survey for pregnant women in antenatal clinics
B	Women who meet the eligibility criteria and are interested in the RCT are visited in their home to obtain formal consent
C	After obtaining formal consent, eligible women will complete a baseline questionnaire
D	Sustained nurse home visiting intervention
E	Study 6 week follow-up to assess pregnancy, health care access and health literacy
F	Study 6 month follow-up to collect new contact details, health literacy and health service use data
G	Study 1 year follow-up to assess primary and secondary outcomes
H	Study 18 month follow-up to collect new contact details, health literacy and health service use data
I	Study 2 year follow-up to assess primary and secondary outcomes

The schedule for collecting primary and secondary outcomes is presented in Table 4 below. These were previously described in Section 4.5. Additional details about these measures and their psychometric properties are provided in Appendix 4.

Table 4: Study measures collected at each time point

	**Time point**					
**Study Measure**	**Enrol**	**6w**	**6m**	**1y**	**18m**	**2y**
Demographics	X	X	X	X	X	X
Contact details	X	X	X	X	X	X
PRIMARY OUTCOMES						
Maternal						
Parent care: Nutrition, Safety, Sleep				X		X
Parent responsivity				X		X
Home Environment:						
Confusion, Hubbub and Order scale (CHAOS)	X			X		X
Home Observation for Measurement of the Environment (HOME) subscales				X		X
MacArthur Communicative Development Inventory (MCDI)						X
SECONDARY OUTCOMES						
Maternal						
Pregnancy Outcomes		X				
Social support	X			X		X
Quality of Life	X	X		X		X
Maternal mental health	X	X		X		X
Parenting Wellbeing	X			X		X
General Health Questionnaire	X	X	X	X	X	X
Parenting self-efficacy	X			X		X
Health service use		X	X	X	X	X
Health literacy			X		X	
Parenting styles	X			X		X
Child						
Mental and general health						X
General Health		X	X	X	X	X
Sibling						

	Time point					
**Study Measure**	**Enrol**	**6w**	**6m**	**1y**	**18m**	**2y**
Mental health/behaviour	X			X		X
Nurse						
Use and knowledge of evidence-based clinical strategies	X	X	X	X	X	X
MONITORING VARIABLES						
Maternal social capital/sensitive issues	X			X		X
Child birth data		X				
Child growth data (e.g. height & weight)		X	X	X	X	X
Intervention dose and fidelity		X	X	X	X	X

Time points: w=week, m=month, y=year. Enrolment will occur within a fortnight of screening at the antenatal hospital clinic. Monitoring items are variables that may be used to describe the sample or to control for potential confounding and are not collected as demographics.

8. CLINICAL ASSESSMENTS

We plan to collect all assessment data electronically via tablets; however, we will print paper versions of all assessments as backup for possible electronic malfunctions.

Screening: The purpose of this face-to-face assessment is to identify eligible women and invite them into the right@home trial. As described in Section 6.1, researchers will approach pregnant women in antenatal clinic waiting rooms using the script in **Appendix 16**. Interested women will be asked to complete informed consent (see **Appendix 17**) and complete the screening survey (see **Appendix 15**), either with the researcher or alone. Using the eligibility algorithm identified by the pilot (RCH HREC # 32271, Northern Health HREC P 20/12), researchers will identify eligible women from their survey responses and invite them into the next phase of the trial. This involves describing RCT participation, giving women the information statement for participation in the randomised controlled trial (**Appendix 2**), recording their contact details and organising a time to visit them in their home to conduct the formal enrolment.

Baseline enrolment: The purpose of this face-to-face assessment is to formally enrol (i.e. obtain informed consent) for participation in the right@home trial, and conduct a comprehensive baseline assessment of women. A researcher will visit the participant in her home (or at The Royal Children's Hospital or a mutually-convenient location nearby, depending on the woman's preference and researcher safety). The researcher will answer any questions the woman has about the Information Statement given to her at the screening assessment and collect informed consent (see **Appendix 17**). The researcher will then conduct the baseline questionnaire with the mother (see Appendix 20). The questionnaire will be collected in a private space, i.e. a separate room with only the mother, the researcher, and any young children that the mother needs to care for, present. The baseline assessment includes the mother completing the SDQ for any children aged 3-6 years (see Sections 4.5 and 7 above). We expect the baseline enrolment assessment will take 50-60 minutes to

complete face-to-face, based on purposeful piloting (i.e. the interviewing was undertaken with the understanding that it would be a slower process than anticipated) with our research staff.

Once the woman completes the baseline assessment, she will be randomised into one of the intervention or usual care arms. Once randomised, the participant's details will be emailed to the right@home nurse responsible for the region, and the nurse will be responsible for initiating contact and beginning the intervention.

9. ADVERSE EVENT REPORTING

9.1 Definitions

Adverse Event (AE): Any untoward medical occurrence in a participant enrolled into this study regardless of its causal relationship to study treatment. Adverse events are classified as serious or non-serious.

Serious Adverse Event (SAE)

An SAE is defined as any AE that:

- results in death; or
- is immediately life threatening; or
- requires inpatient hospitalisation; or
- requires prolongation of existing hospitalisation; or
- results in persistent or significant disability/incapacity; or
- is a congenital anomaly/birth defect.

Important medical events will be considered an SAE when, based upon appropriate medical judgement, they may jeopardize the patient and may require medical or surgical intervention to prevent one of the outcomes listed in this definition.

We recognise that participation in the right@home study may be a cause of potential stress or harm to participants, if it asks questions that are distressing, or if participation leads to disagreements or fights between the participant and her partner and/or family. Potential stress will be minimised in the following ways:

1. The participant will be fully aware of the reason for the questions asked in the assessments
2. The participant will be advised that they can decline to continue in the trial at any time
3. Participants will be offered appropriate referral for any concerns raised

At recruitment screening, any women who becomes distressed will be encouraged to discuss their concerns with the health provider they are about to see. During the study if a research assistant is

concerned about a parent they will discuss this with the PI. With the parent's permission a referral mechanism will be in place to the parent's local doctor or maternal and child health nurse. This process has been implemented successfully with a number of other studies that specifically ask about self harm behaviour. If the parent is within the intervention arm the PI will contact the parent and ask permission to refer back to the intervention nurse.

At the 6 week, 6 month and 18 month follow-up phone assessments, women will be asked what things they like and dislike about being part of the study, and whether being part of the study has caused them any harm so far. These questions will be developed based on previous MCRI studies that have successfully used these processes (e.g. Weave Study, Healthy Mothers Study) and submitted for approval in a future ethics modification.

Participants will be advised that non-participation in the trial will not affect their access to routine antenatal, postnatal and early childhood clinical management and services. Participants will, at a minimum, receive usual clinical management in accordance with Victorian and Tasmanian universal antenatal, postnatal and child and family management protocols. Participation in the research will not adversely affect the participants – it is hypothesised that participants in the intervention group will show improved child and maternal outcomes.

9.2 Assessment and Documentation of Adverse Events

For the purposes of this study the investigator is responsible for recording all Adverse Events, regardless of their relationship to the study intervention, with the following exceptions:

- Conditions that are present at screening and do not deteriorate will not be considered adverse events.

The description of each AE on the study database will include:

- A description of the AE;
- The onset date, duration, date of resolution;
- Severity (mild, moderate or severe);
- Seriousness (i.e. is it an SAE?);
- Any action taken, (e.g. treatment, follow-up tests);
- The outcome (recovery, death, continuing, worsening);
- The likelihood of the relationship of the AE to the study treatment (Unrelated, Possible, Probable, Definite).

The seriousness of an AE will be assessed by an investigator according to the definition in section 9.1, with the following exception:

- Hospitalisation due to progression of disease will not be considered an SAE for the purposes of this study.

For the right@home study, the most likely AEs/SAEs that nurses and researchers will encounter are psychosocial, substance abuse, home safety and child protection issues (e.g. abuse, neglect). Where an incident that requires a child protection notification occurs during a nurse visit, the nurses will respond in accordance with their local mandatory reporter guidelines. This is part of usual care, and thus involves no change in practice for the nurses. The baseline PIS&C (Appendix 2) includes the following: “We can disclose the information only with your permission, unless required by law”, which alerts the participant to the need to remain within the law.

Where the incident occurs during a researcher visit, we will follow the processes used successfully in MECSH. For psychosocial and drug/alcohol problems, researchers will recommend that women phone the maternal and child health line (13 22 29). They will also ask if the woman would like CI Goldfeld to phone and offer a more specific referral. For domestic violence and child abuse/neglect problems, researchers will report using the local public child protection system, i.e. via the usual public child protection helpline (132 111). Researchers with concerns will be asked to discuss, in the first instance, the child protection issue with CI Goldfeld (see details in the Safety and Communication Protocol, Appendix 21). While researchers are not mandatory reporters, CI Goldfeld is. The research team will follow-up as required based on the response from the child protection system. The research staff will also be provided with support and debriefing from CIs and MCRI counselling.

Changes in the severity of an AE will be reported. AEs characterized as intermittent will be documented for each episode. All AEs will be followed to adequate resolution, where possible.

9.3 Eliciting Adverse Event Information

Adverse events will be recorded from the time the patient signs the informed consent form until 30 days after the last intervention nurse visit. At every study visit patients will be asked “How have you felt since your last visit?” in order to elicit any medically related changes in their well-being. They will also be asked if they have been hospitalised, had any accidents, used any new medication or changed concomitant medication regimens. In addition, AEs will be documented from physical examination findings, clinically significant lab results or other documents (including patient diaries and correspondence from their primary care physician) that are relevant to patient safety.

9.4 Serious Adverse Event Reporting

9.4.1 SAEs

Any SAE occurring in a study participant will be reported to the RCH HREC within 24-72 hours of occurrence, in accordance with the safety reporting policy of the HREC. The HREC safety reporting form will be completed, signed and submitted by an investigator.

10. STATISTICAL METHODS

10.1 Sample Size Estimation

The sample size calculation is bound by two factors. First, we are attempting to increase the apparent effectiveness of SNHV compared with other randomised controlled trials (RCTs) for child and parent outcomes. Despite their intensity, existing trials like MECSH show relatively modest effects (effect sizes of 0.2-0.4 standard deviations).[4, 7] Effect sizes of 0.25-0.3 SDs can be meaningful at the public health level where the effect is diluted over a population. However, at a clinical level, an effect size of 0.2 is typically deemed small; 0.5 is medium; and 0.8 is large[14]. In the case that the right@home SNHV program is not effective, we will need to be able to detect the modest effect sizes found in earlier studies. Interestingly, previous home visiting randomised controlled trials rarely conducted sample size calculations and, as very few examine the same primary outcomes as right@home, there is no comparison for choosing a sample size. The second factor is that the sample size is constrained by the availability of funding for the SNHV services. The maximum number of full-time nurses we can have on the study is 10 in Victoria and 4 in Tasmania, which means the maximum number of intervention participants (an average of 29 per nurse) is n=400.

Randomisation of 400 mothers per arm is required to provide 80% power to detect a minimum difference of 0.315 standard deviations on any continuous outcome measure, allowing for an average intra-class correlation coefficient of 0.02 and an average cluster size of 29, to estimate relatedness of maternal and child outcomes within each nurses case allocation. This sample size also allows for a retention rate of 60% of mothers until the assessment of the primary outcome at age 2 years. The study sample size was estimated using the Stata software package. [Stata Statistical Software: Release 11.1. College Station, TX: StataCorp LP; 2011.] Depending on recruitment rates and study progress, there may be a sample size review over the course of the trial.

We acknowledge that our choice of a 40% attrition rate is lower than the attrition in MECSH (45% participated in 2 year follow-up in the control arm compared with 57% in the intervention arm). This is for two reasons. First, in MECSH, participants were lost to follow-up if they changed addresses out of the single Sydney suburb that the trial was conducted in. We expect the right@home families to be equally transient, but the nurses and researchers can still reach them if they live in the same region. There is also the possibility that families could swap nurses if they move out of one right@home region and into another, and this will be organised if and when cases arise. As such, we are confident that we will lose fewer families to itinerancy. Second, according to Lynn Kemp, part of the MECSH attrition was due to families not being high-risk enough to benefit from the program and feeling that they did not need the information. This is supported by the home visiting literature which suggests that the most vulnerable families benefit most from the interventions.[4] For this reason, right@home aims to recruit only the most vulnerable families (i.e. ~5% rather than ~15% in MECSH).

We acknowledge that differential attrition, as evident in the MECSH follow-up, is a significant risk. The primary reason for the follow-up phone calls to all families at 6 weeks, 6 and 18 months is to maintain contact. The secondary reason is to collect data so that the burden of the face-to-face assessments is reduced. We will contact the right@home nurses and maternal and child health nurses responsible for the usual care families for contact details when we lose contact. If it is

possible, we will obtain consent to record the family's Centrelink number so that we can obtain current contact details if we lose contact with the family. We are currently making inquiries into whether this is possible and will only be able to do it with the correct authority and ethical approval.

10.2 Population to be analysed

The intention-to-treat population will be used in the analyses. Mothers (and children) will be compared according to the group to which they were randomly allocated, regardless of mothers' compliance, crossover to other treatments or withdrawal from the study. This approach preserves the prognostic balance in the study arms achieved by randomisation.

10.3 Statistical Analysis Plan

The frequency and patterns of missing data will be examined and sensitivity analyses will be performed comparing the results of analyses restricted to families with complete data and analyses where missing data are imputed using a conservative approach.[15]

The baseline characteristics of the mothers will be presented for each treatment arm using the mean, median and IQR for continuous data and proportions for categorical data. Maternal and child outcome measures will be similarly described by treatment arm and comparisons made using the t-test and non-parametric tests for continuous data and the chi-squared test for categorical data.

Linear and logistic regression modelling will be conducted to test the primary and secondary hypotheses listed below, taking into account maternal baseline and child characteristics identified *a priori* i.e. child gender, socio-demographic characteristics of the mother and family, and maternal specific characteristics including health, mental health and self efficacy.

<u>Objectives/hypotheses:</u>

Primary: At child age 2 years, compared with the usual care group, intervention mothers will demonstrate:

1. Improved parent care, i.e. the parent's ability to provide a consistent, regular and supportive environment for their child;
2. Improved parent responsivity, i.e. the parent's ability to tune in to their child's needs and to respond appropriately; and
3. A more supportive home environment, i.e. building a strong home learning environment through structured developmental promotion activities focusing on language.

Secondary: At child age 2 years, compared with the usual care group, intervention mothers will demonstrate:

1. Maternal: Improved pregnancy outcomes, Quality of Life, Maternal mental health, Parent wellbeing, General Health, Parenting self-efficacy, Health literacy, and more effective Health service use. The latter will depend on the service, e.g. preventative healthcare and

management vs. emergency department visits, and will be determined in partnership with the study health economists.

2. Child: Improved mental and general health, and language
3. Sibling: Improved mental health/behaviour
4. Nurse: Increased use and knowledge of evidence-based strategies

We have not powered the trial to consider subgroups but there will be some analyses which will need to be restricted to a subgroup of the trial, e.g. anything that refers to current parenting attitudes or behaviours (as not all women will already have children). These subgroup analyses will only be powered for larger effect sizes than the primary outcomes, and will be provided in a future ethics modification once the final outcome measures are decided. Another subgroup analyses which the team may consider (and, again, power will be limited) is testing whether the findings appear to be consistent by site/state.

10.4 Interim Analyses

No interim analyses will be conducted for this study.

11. DATA MANAGEMENT

11.1 Data Collection

All assessments will be conducted face-to-face or over the phone, via interview. All questionnaires will be developed to be collected electronically (on tablets) such that accidentally missing questions will not be possible. Women will be able to voluntarily skip questions which will require an active override by the research assistant. This means that no electronically-collected data will be truly missing unless a woman is unable to complete an assessment for some reason. Paper versions of assessments will be provided in the case of electronic/technical malfunction. Again, these will be collected via interview so questions will not be missing unless the woman chooses not to answer them. On completing assessments, data will be uploaded from the tablet (or paper version) to the study database through a secure connection.

11.2 Data Storage

All participants and nurses will be given unique numerical identifiers (an ID code) for use throughout the study. A single, online electronic database will record all participant and nurse details. It will be hosted by the MCRI server, because it is secure and meets ethical confidentiality requirements. Nurses, researchers and managerial staff will have different levels of access to the password-protected database. Nurses will only be able to access the details of the families in their caseload; researchers will be able to access the details of families but not their randomisation status; and managerial staff will be able to access all levels as required. All participant and nurse questionnaire data will be identified by ID code only and be stored in the secure electronic database. Digital video

material (i.e. collected by intervention nurses to conduct video feedback with families) will be attached as electronic files to the family's record in the database. Any written materials will be scanned and also saved with the family's record on the electronic database. Hardcopy (paper) nurse materials will be stored in a locked cabinet at CHETRE, and hardcopy family materials will be stored in a locked cabinet at CCCH. Only project staff directly involved in the analysis of the data will have access to these materials and the electronic database. The database linking the participants' identity to their ID code will be maintained electronically on a password protected file.

11.3 Study Record Retention

All project materials will be stored on the password-protected electronic database or in locked cabinets for the required period of time, i.e. indefinitely if the participant consents to providing their data for data pooling or, otherwise, until the youngest participant is 25-years-old, e.g. until July 31st 2029. After that time, hardcopy materials will destroyed by shredding, and any password protected electronic archives will be permanently deleted.

12. ADMINISTRATIVE ASPECTS

12.1 Confidentiality

Subject confidentiality is strictly held in trust by the participating investigators, research staff, and the sponsoring institution and their agents, and is extended to cover clinical information relating to participating subjects. The study protocol, documentation, data and all other information generated will be held in strict confidence. No information concerning the study or the data will be released to any unauthorized third party, without prior written approval of the sponsoring institution. The HRECs of the sponsoring institution may inspect all documents and records required to be maintained by the Investigator, including but not limited to, medical records (office, clinic or hospital) and pharmacy records for the subjects in this study. The clinical study site will permit access to such records. All evaluation forms, reports and other records that leave the site will be identified only by the Subject Identification Number to maintain subject confidentiality. Clinical information will not be released without written permission of the subject, except as necessary for monitoring by HREC or regulatory agencies

12.2 Independent HREC Approval

This protocol and the informed consent document and any subsequent modifications will be reviewed and approved by the human research ethics committee (HREC). A letter of protocol approval by HREC will be obtained prior to the commencement of the study, as well as approval for other study documents subject to HREC review.

12.3 Modifications of the protocol

This study will be conducted in compliance with the current version of the protocol. Any change to the protocol document or Informed Consent Form that affects the scientific intent, study design, patient safety, or may affect a participants willingness to continue participation in the study is considered an amendment, and therefore will be written and filed as an amendment to this protocol and/or informed consent form. All such amendments will be submitted to the HREC, for approval prior to becoming effective.

12.4 Protocol Deviations

All protocol deviations must be recorded in the patient record (source document) and on the CRF and must be reported to the PI. Protocol deviations will be assessed for significance by the Principal Investigator. Those deviations deemed to have a potential impact on the integrity of the study results, patient safety or the ethical acceptability of the trial will be reported to the HREC during the course of the study from 2013-16. Where deviations to the protocol identify issues for protocol review, the protocol will be amended as per section 12.3

12.5 Participant Reimbursement

In this study each participant will be required to make time for 3 face-to-face assessments and 3 phone assessments. This is a relatively significant commitment for participants. Participants will not be fully reimbursed for their time; however, a gift voucher to the value of at least $20 and no more than $30 for use at places such shopping centres (which prohibit the purchase of alcohol), cinemas, and other family-friendly activities or toys will be provided as a token of appreciation at each face-to-face assessment. Based on the findings from the recruitment pilot, $20-$30 was the preferred voucher amount (preferred by 40% of participants; however, 21% of women did not think the amount mattered). Participants will be informed of this token voucher at the outset.

12.6 Financial Disclosure and Conflicts of Interest

The investigators have no conflicts of interest to declare.

13. USE OF DATA AND PUBLICATIONS POLICY

Please see the study team’s Publication MOU in Appendix 22.

14. LIST OF ATTACHED APPENDICES

Appendix 1: Expert Reference Group Terms of Reference

Appendix 2: right@home Participant Information and Consent for RCT

Appendix 3: CCCH home visiting literature review

Appendix 4: Table of baseline measures and their psychometric properties

Appendix 5: MECSH program manual for nurses

Appendix 6: right@home draft addendum for MECSH program

Appendix 7: right@home online training program summary (first six modules)

Appendix 8: MECSH Nurse antenatal fidelity checklist

Appendix 9: MECSH Nurse postnatal fidelity checklist

Appendix 10: Introductory email to Vic antenatal managers

Appendix 11: Introductory email to Tas antenatal managers

Appendix 12: One-page information sheet for clinic staff

Appendix 13: Clinic poster

Appendix 14: right@home flyer for parents

Appendix 15: right@home Screening survey

Appendix 16: right@home Screening script

Appendix 17: right@home Participant Information and Consent for Screening survey

Appendix 18: right@home participant contact details slip

Appendix 19: right@home study contact details card

Appendix 20: right@home Baseline enrolment questionnaire

Appendix 21: right@home Safety and Communication Manual

Appendix 22: right@home draft Publications guidelines

Appendix 23: Complete risk management plan

Appendix 24: Partnership agreement/contract between ARACY, CCCH and CHETRE

Appendix 25: Local council contract agreements: Whittlesea Agreement

Appendix 26: Local council contract agreements: Whittlesea Signatures

Appendix 27: Local council contract agreements: Ballarat

Appendix 28: Local council contract agreements: Dandenong

Appendix 29: Local council contract agreements: Tasmania

Appendix 30: CCCH Focus Modules Literature Review

Appendix 31: Victorian Specific Module

Appendix 32: Scientific review responses

15. REFERENCES

1. Brooks-Gunn, J., & Duncan, G.J. (1997). The effects of poverty on children. Future of Children, 7, 55-71; Duncan, G. J. & Brooks-Gunn, J. (2000). Family poverty, welfare reform and child development. Child Development, 71, 188-196; Ryan, R. M., Fauth, R. C., & Brooks-Gunn, J. (2006). Childhood poverty: Implications for school readiness and early childhood education. In B. Spodek & O. N. Saracho (Eds.), Handbook of research on the education of young children (2nd ed., pp. 323–346). Hillsdale, NJ: Erlbaum.
2. Watson J, Tully L. Prevention and Early Intervention Update – Trends in Recent Research. Sydney: NSW Department of Community Services, Centre for Parenting and Research; 2008.
3. Watson J, White A, Taplin S, Huntsman L. Prevention and Early Intervention Literature Review. Sydney: NSW Department of Community Services; 2005.
4. McDonald M, Moore T, Goldfeld S. Sustained Home Visiting for Vulnerable Families and Children: A Literature Overview. Melbourne: Centre for Community Child Health (CCCH) & Murdoch Children's Research institute (MCRI); 2012.
5. Nurse-Family Partnership. Undated. It's about changing lives [Brochure]. Colorado, Nurse Family Partnership. Accessed 27/06/2012 from http://www.nursefamilypartnership.org/assets/PDF/News/NFP-overview_brochure-single_pages. In.
6. Kemp L, Harris E, McMahon C, et al. Miller Early Childhood Sustained Home-visiting (MECSH) trial: design, method and sample description. *BMC Public Health* 2008;8:424.
7. Kemp L, Harris E, McMahon C, et al. Child and family outcomes of a long-term nurse home visitation programme: A randomised controlled trial. *Archives of Disease in Childhood* 2011;96(6):533-540
8. For example,Victoria's Vulnerable Children Policy (see http://www.dhs.vic.gov.au/about-the-department/news-and-events/news/general-news/victorias-vulnerable-children--our-shared-responsibility) and National Framework for Protecting Australia's Children (http://www.communities.qld.gov.au/resources/childsafety/child-protection/national-framework.pdf). In.
9. Farah MJ, Ferrari M, Vuletic L. Mind, Brain, and Education in Socioeconomic Context. In: Developmental Relations among Mind, Brain and Education: Springer Netherlands; 2012. p. 243-256.
10. Segal L, Sara Opie R, Dalziel KIM. Theory! The Missing Link in Understanding the Performance of Neonate/Infant Home-Visiting Programs to Prevent Child Maltreatment: A Systematic Review. *Milbank Quarterly* 2012;90(1):47-106.
11. Marmot M, Allen J, Goldblatt P, Boyce T, McNeish D, Grady M. Fair society, healthy lives: the Marmot review; strategic review of health inequalities in England post-2010: Marmot Review; 2011.
12. Moore T, McDonald M, Sanjeevan S. Evidence-based service modules for a sustained nurse home visiting program: A literature review Melbourne: Centre for Community Child Health (CCCH) & Murdoch Children's Research institute (MCRI); 2012.
13. Chittleborough CR, Lawlor, Debbie A., Lynch, John W. Young maternal age and poor child development: predictive validity from a birth cohort. *Pediatrics* 2011;127(6):e1436-44.
14. Cohen J. A power primer. *Psychol Bull* 1992;112(1):155-159.
15. Sterne JAC, White IR, Carlin JB, et al. Multiple imputation for missing data in epidemiological and clinical research: potential and pitfalls. *BMJ: British Medical Journal* 2009;338.
