# Supplementary material for: Nurse home visiting to improve child and maternal outcomes: 5-year follow-up of an Australian randomised controlled trial
Source: PLoS One. 2022 Nov 28;17(11):e0277773. doi: 10.1371/journal.pone.0277773 (PMC9704648; doi:10.1371/journal.pone.0277773)
Supplement: Attachment — Submitted filename: renamed_519c7.docx [file pone.0277773.s007.docx]

**COMMENTS – Manuscript PONE-D-22-13241**

Title: “Nurse home visiting to improve child and maternal outcomes: 5-year follow-up of an Australian randomised controlled trial.”

**Important note: This review pertains only to ‘statistical aspects’ of the study and so** **‘clinical aspects’ [like medical importance,** **relevance of the study,** **‘clinical significance and implication(s)’ of the whole study, etc.] are to be evaluated [should be assessed] separately/independently. Further please note that any ‘statistical review’ is generally done under the assumption that (such) study specific methodological [as well as execution] issues are perfectly taken care of by the investigator(s). This review is not an exception to that and so does not cover clinical aspects {however, seldom comments are made only if those issues are intimately / scientifically related & intermingle with ‘statistical aspects’ of the study}. Agreed that ‘statistical methods’ are used as just tools here, however, they are vital part of methodology [and so should be given due importance].**

**COMMENTS:** It must be appreciated that the study is excellently planned and executed. There are hardly any issues, the only comment is: no reference is provided for [line 242] Socio-Economic Index for Areas (SEIFA).

However, I have two small suggestions (for kind consideration of authors) as follows:

- To provide a description of baseline characteristics is entirely reasonable (since it is clearly important in assessing to whom the results of the trial can be applied), however, statistical comparison of baseline characteristics [last ‘p-value’ column in Table 2] is not desirable at all [because even if P-value turns out to be significant (while comparing baseline characteristics despite random allocation), it is, by definition, a false positive] as you then are supposed to be testing ‘randomization’ then, which in any single trial may not balance all baseline characteristics [Participants were then randomised to control or intervention arms, lines 171-2] because ‘randomization’ is a sort of ‘insurance’ and not a guarantee scheme.

References:

1. Stuart J. Pocock, et al., ‘Subgroup analysis, covariate adjustment and baseline comparisons in clinical trial reporting: current practice and problems’, Statistics in medicine, 2002; 21:2917–2930 [Particularly page 2927]
2. Harrington D, et al., ‘New guidelines for statistical reporting in the journal’, N Engl J Med 2019;381:285-6

[Important message (indirectly/ultimately indicated) from these articles: Never do any comparison with respect to ‘baseline’ characteristics {by applying statistical significance test(s)}, when allocation is done randomly].

- Though the measures/tools used are appropriate {like Depression, Anxiety and Stress Scales, Personal Wellbeing Index, or Assessment of Quality of Life – 8D (AQoL-8D)} most of them yield data that are in [at the most] ‘ordinal’ level of measurement [and not in ratio level of measurement for sure {as the score two times higher does not indicate presence of that parameter/phenomenon as double (for example, a Visual Analogue Scales VAS score or say ‘depression’ score)}]. Then application of suitable non-parametric test(s) is/are indicated/advisable [even if distribution may be ‘Gaussian’ (i.e. normal)]. Agreed that there is/are no non-parametric test(s)/technique(s) available to be used as alternative in all situation(s) [suitable / most desired/applicable], but should be used whenever/wherever they are available. [Why not direct comparison and not comparison through regression which is indirect as regression techniques are not originally developed for comparison?]

Moreover, I would like to ask two questions :

- What is BMJ’s patient partnership strategy (line 153)?
- According to lines 226-7 [At 4 and 5 years, 64·4% and 59·0% participants provided data respectively] do not you think that drop-outs are too much {though its ‘retaining the power of the original sample size calculation & using a combination of multiple imputation (52) and inverse probability weighting (53) to account for missing data amongst respondents as per lines 235-237}?

This manuscript is alright otherwise. We can accept it after minor revision, in my opinion.
