# Supplementary material for: Nurse home visiting to improve child and maternal outcomes: 5-year follow-up of an Australian randomised controlled trial
Source: PLoS One. 2022 Nov 28;17(11):e0277773. doi: 10.1371/journal.pone.0277773 (PMC9704648; doi:10.1371/journal.pone.0277773)
Supplement: Attachment — Submitted filename: Response to Reviewers (PONE-D-22-13241).docx [file pone.0277773.s008.docx]

To the Academic Editor: Dr. Andrea Martinuzzi

8^th^ September 2022

Dear Dr. Andrea Martinuzzi,

Please find enclosed our revised Manuscript PONE-D-22-13241 “**Nurse home visiting to improve child and maternal outcomes: 5-year follow-up of an Australian randomised controlled trial**”.

We note the positive comments of the Reviewers, which highlight the rigorous study design and execution. We have carefully considered and addressed all comments and hope you find the paper to be strengthened in clarity and quality.

We believe that this paper makes important contributions to both the nurse home visiting and health equity literature. It provides robust evidence generated in real world health care systems and supports nurse home visiting as a potential platform to achieve substantial and equitable benefits to women’s and children’s health and wellbeing.

With regards to Data Availability, data are fully available to researchers who request access to the data from the Melbourne Children's Campus LifeCourse institutional data access platform (<https://lifecourse.melbournechildrens.com/data-access/>). Due to the type of ethical approval (for sensitive information), data sharing is subject to use for research processes and cannot be published on a public repository. This Data Availability statement has been accepted for a recently published PLOS ONE manuscript that drew on the same right@home dataset, written by some of the same authors as this submission (Bryson H, Mensah F, Price A…Goldfeld S. (2021) Clinical, financial and social impacts of COVID-19 and their associations with mental health for mothers and children experiencing adversity in Australia. PLOS ONE 16(9): e0257357; https://doi.org/10.1371/journal.pone.0257357). Given the PLOS ONE Criterion 3 asks “Have the authors made all data underlying the findings in their manuscript fully available?” and the response options are binary (yes/no), we have included this information in the cover letter, the response to reviewers, and the manuscript to ensure this nuance is captured.

We are happy to consider additional changes at your request.

Yours sincerely,

Prof Sharon Goldfeld.

**PONE-D-22-13241 - Response to Editorial and Reviewer comments**

| **Comment** | **Response** |
| --- | --- |
| **Editor** |  |
| Please ensure that your manuscript meets PLOS ONE's style requirements, including those for file naming. | We confirm that the revised manuscript meets PLOS ONE’S style requirements. |
| Thank you for submitting your clinical trial to PLOS ONE and for providing the name of the registry and the registration number. The information in the registry entry suggests that your trial was registered after patient recruitment began. PLOS ONE strongly encourages authors to register all trials before recruiting the first participant in a study.  As per the journal’s editorial policy, please include in the Methods section of your paper: (1) your reasons for your delay in registering this study (after enrolment of participants started);  (2) confirmation that all related trials are registered by stating: “The authors confirm that all ongoing and related trials for this drug/intervention are registered”. | (1) Thank you for noting the registration. We applied to register the trial with ISRCTN in 2013 before recruitment commenced. However, a year after it was registered, it was labelled ‘retrospective’ in 2014. The allocation was a new field and automatically generated based on when funds were paid and not registration per se. At no time was any of this information available when we were registering in 2013. Our timeline meets the definition of prospective research used by the International Committee of Medical Journal Editors, which states that: “*ICMJE uses the date trial registration materials were first submitted to a registry as the date of registration. When there is a substantial delay between the submission of registration materials and their posting at the trial registry, editors may inquire about the circumstances that led to the delay*” (<http://www.icmje.org/recommendations/browse/publishing-and-editorial-issues/clinical-trial-registration.html>). In our case the date was 16 April 2013 (date applied for registration with ISRCTN). All approvals for human research were obtained by 14 March 2013. The first participant was enrolled in the trial on 9 May 2013, thus satisfying the requirements for a ‘prospectively registered’ trial. The trial website declined to change the label but has added editorial notes at the bottom of the trial webpage to provide further information (detailed below and in the cover letter).  *Trial registry editorial notes*  12/12/2017: Recruitment start date corrected from 30/04/2013 to 09/05/2013. Details added to plain English summary, sponsor, funders. New contact. 30/10/2017: This registration was initiated on 16/04/2013 (date applied) and finalised on 21/08/2013 (date payment was received for registration). Relevant ethics approvals were granted before recruitment started on all sites. Recruitment commenced on 30/04/2013 after initiation of public registration. The ISRCTN prospective/prospective flag compares the date of registration payment with the recruitment start date, is automatically generated and does not include any grace period. The flag was introduced in November 2014 (after this trial was registered) and applied to all records in the registry retrospectively using the date information available at the time. The registration of this study was finalised within 6 months of the recruitment starting. 17/10/2017: IPD sharing statement added. 23/03/2017: Publication reference added. 07/10/2016: The interventions, outcomes and exclusion criteria have been updated. 27/07/2016: the participant exclusion criteria has been updated. 25/07/2016: The publication and dissemination plan and availability of the participant level data have been added. 15/07/2016: The recruitment start date has been updated from 01/04/2013 to 30/04/2013. 24/09/2015: The recruitment end date was changed from 31/12/2017 to 31/08/2015. 04/07/2014: The target number of participants was changed from 800 to 714.  (2) There are no ongoing trials currently related to right@home. We have added the following to this section:  *No other trials of the right@home intervention are ongoing*. |
| We note that you have stated that you will provide repository information for your data at acceptance. Should your manuscript be accepted for publication, we will hold it until you provide the relevant accession numbers or DOIs necessary to access your data. If you wish to make changes to your Data Availability statement, please describe these changes in your cover letter and we will update your Data Availability statement to reflect the information you provide | With regards to Data Availability, data are fully available to researchers who request access to the data from the Melbourne Children's Campus LifeCourse institutional data access platform (https://lifecourse.melbournechildrens.com/data-access/). Due to the type of ethical approval (for sensitive information), data sharing is subject to use for research processes and so cannot be published on a public repository. This Data Availability statement has been accepted for a recently published PLOS ONE manuscript that drew on the same right@home dataset, written by some of the same authors as this submission (Bryson H, Mensah F, Price A…Goldfeld S. (2021) Clinical, financial and social impacts of COVID-19 and their associations with mental health for mothers and children experiencing adversity in Australia. PLOS ONE 16(9): e0257357 https://doi.org/10.1371/journal.pone.0257357). We note this in each of the cover letter, the response to reviewers, and the manuscript, because this nuance has previously been missed at the review stage, i.e. the PLOS ONE Criterion 3 check box asks “Have the authors made all data underlying the findings in their manuscript fully available?” and the response options are binary (yes/no).  The updated data statement is:  *We invite researchers to request access to the data from the Melbourne Children's Campus LifeCourse institutional data access platform (*[*https://lifecourse.melbournechildrens.com/data-access/*](https://lifecourse.melbournechildrens.com/data-access/)*). Data are not published in a public repository because they contain sensitive participant information which are restricted to use for research purposes only. This restriction is in accordance with the Participant Information and Consent Form and approved study protocol, governed by the Royal Children’s Hospital Human Research Ethics Committee (HREC 32296)*. Related documents, such as the study protocol, statistical analysis plan, informed consent form, are also available on request. |
| We note that you have provided funding information that is not currently declared in your Funding Statement. However, funding information should not appear in the Acknowledgments section or other areas of your manuscript. We will only publish funding information present in the Funding Statement section of the online submission form.  Please remove any funding-related text from the manuscript and let us know how you would like to update your Funding Statement. | As requested, the acknowledgements section has been removed from the manuscript. All funding information is included in the ‘Funding Statement’ section of the manuscript; this corresponds to the funding information section on the submission portal.  Please update the funding statement as follows:  *This work was supported by the state governments of Victoria and Tasmania, the Ian Potter Foundation, Sabemo Trust, Sidney Myer fund, the Vincent Fairfax Family Foundation, and the National Health and Medical Research Council (NHMRC, 1079148). The Murdoch Children’s Research Institute administered the research grant for the study and provided infrastructural support to its staff but played no role in the conduct or analysis of the trial. Research at the MCRI is supported by the Victorian Government's Operational Infrastructure Support Program. SG was supported by NHMRC Practitioner Fellowship (1155290). FM was supported by NHMRC Career Development Fellowship (1111160)*. |
| **Reviewer 1** |  |
| Important note: This review pertains only to ‘statistical aspects’ of the study and so ‘clinical aspects’ [like medical importance, relevance of the study, ‘clinical significance and implication(s)’ of the whole study, etc.] are to be evaluated [should be assessed] separately/independently. Further please note that any ‘statistical review’ is generally done under the assumption that (such) study specific methodological [as well as execution] issues are perfectly taken care of by the investigator(s). This review is not an exception to that and so does not cover clinical aspects {however, seldom comments are made only if those issues are intimately / scientifically related & intermingle with ‘statistical aspects’ of the study}. Agreed that ‘statistical methods’ are used as just tools here, however, they are vital part of methodology [and so should be given due importance]. | Thank you for your thorough statistical review of the manuscript. |
| 1. It must be appreciated that the study is excellently planned and executed. There are hardly any issues, the only comment is: no reference is provided for [line 242] Socio-Economic Index for Areas (SEIFA). | Thank you. As requested, we have added a reference for SEIFA (see below).  Treatment effects were estimated using regression methods (linear or logistic models), adjusted for the stratification factors used during randomisation: parity and study site; and baseline covariates: family’s Socio-Economic Index for Areas (SEIFA) score (55) indicating neighbourhood level disadvantage, maternal education, maternal age at child’s birth, parity, antenatal risk, maternal self-efficacy, and maternal mental health, as well as child sex and age at the 4- and 5-year assessments. |
| 2. To provide a description of baseline characteristics is entirely reasonable (since it is clearly important in assessing to whom the results of the trial can be applied), however, statistical comparison of baseline characteristics [last ‘p-value’ column in Table 2] is not desirable at all [because even if P-value turns out to be significant (while comparing baseline characteristics despite random allocation), it is, by definition, a false positive] as you then are supposed to be testing ‘randomization’ then, which in any single trial may not balance all baseline characteristics [Participants were then randomized to control or intervention arms, lines 171-2] because ‘randomization’ is a sort of ‘insurance’ and not a guarantee scheme. | The p values had been included to reflect the differences that may have arisen due to selective attrition rather than the baseline randomisation specifically. However, we had specified a priori that any differences that had arisen due to attrition would be addressed by the implementation of Inverse Probability Weighting in combination with multiple imputation to address missed data items. As suggested by the Reviewer, the p-values have been removed from Table 2 (baseline characteristics). |
| 3. Though the measures/tools used are appropriate {like Depression, Anxiety and Stress Scales, Personal Wellbeing Index, or Assessment of Quality of Life – 8D (AQoL-8D)} most of them yield data that are in [at the most] ‘ordinal’ level of measurement [and not in ratio level of measurement for sure {as the score two times higher does not indicate presence of that parameter/phenomenon as double (for example, a Visual Analogue Scales VAS score or say ‘depression’ score)}]. Then application of suitable non-parametric test(s) is/are indicated/advisable [even if distribution may be ‘Gaussian’ (i.e. normal)]. Agreed that there is/are no non-parametric test(s)/technique(s) available to be used as alternative in all situation(s) [suitable / most desired/applicable], but should be used whenever/wherever they are available. [Why not direct comparison and not comparison through regression which is indirect as regression techniques are not originally developed for comparison?] | We agree with the Reviewer about the ordinal nature of psychometric measures – compared to measures such as height or weight which have the natural continuity and the ratio level the Reviewer refers to (e.g. 2cm is twice as long as 1cm). However, we have retained the analyses as we note that there is utility in comparing measures to indicate whether there is a difference that is potentially clinically meaningful (e.g. Lumley, T., Diehr, P., Emerson, S. and Chen, L., 2002. The importance of the normality assumption in large public health data sets. Annual review of public health, 23(1), pp.151-169.)  We are unsure about the meaning of the final comment in square brackets. Please let us know if it is outstanding after these revisions. |
| 4. What is BMJ’s patient partnership strategy (line 153)? | Thank you for the note. It was a typographical error and has been removed from the manuscript. |
| 5. According to lines 226-7 [At 4 and 5 years, 64·4% and 59·0% participants provided data respectively] do not you think that drop-outs are too much {though its ‘retaining the power of the original sample size calculation & using a combination of multiple imputation (52) and inverse probability weighting (53) to account for missing data amongst respondents as per lines 235-237}? | When compared with traditional longitudinal studies that include more affluent families, this dropout rate may seem high. However, when compared with nurse home visiting trials that prioritise women and families experiencing adversity, the retention rate is high. For context – as written in the Discussion – by the 2-year follow-up, the UK Building Blocks study retained 71% of their cohort for self-reported outcomes, Pro Kind retained less than 50%, and the French CAPEDP retained only 31% of their original cohort.  With regards to the original power calculations – these assumed attrition of 40% at the primary endpoint of 2 years. We say “retaining the power of the original sample size calculation”, because this level of attrition was only reached at the 5 year follow-up. We note, however, the uncertainty reflected in the confidence intervals, which the retained sample size has contributed to. As there is no distinct point at which the drop-out becomes too much, we interpreted the estimates and confidence intervals as observed and then considered how replicable these are likely to be and how much weight of evidence can be inferred.  The statistical analysis section is revised as follows:  “The original RCT sample size was calculated to detect a minimum effect size (ES) of 0·3 SD in the continuous parent responsivity outcome at the 2-year primary endpoint, *and was based on previous NHV trials.* (52, 53) A target sample size of 714 participants was estimated to provide 80% power, with 5% significance level, accounting for clustering (non-independence of outcomes by the nurse providing NHV or site of usual care provision), and for 40% attrition. A final sample size of 722 participants was achieved. *Retention over time was higher than anticipated and higher than comparable NHV trials. (52, 53)* At 2 years, 558/722 (77·3%) re-enrolled in extended follow-up. At 4 and 5 years, 64·4% and 59·0% participants provided data respectively (detailed in the Results). *As the attrition at 5 years (41%) was that originally calculated for the 2-year primary endpoint, the available dataset retained the power of the original sample size calculation*.” |
| **Reviewer 2** |  |
| 6. The authors present analysis of 4 and 5 year follow-up of a Nurse Home Visiting (NHV) RCT in Australia.   They present results on 29 child outcomes and 26 maternal outcomes. The abstract reports an "overall pattern favouring the intervention" with "statistical evidence of benefits" found across child and maternal mental health and well-being, parenting and family relationships with effect sizes from 0.01-0.27. The analytic strategy is sophisticated and in line with best practice | Thank you. |
| 7. Pg 12. describes original power calculations for a 2 year primary outcome at N= 714 with 722 randomized. Given attrition, it is not clear how power of the original sample size calculation was "retained" at 4-5 years given N=465 at 4 and 426 at 5? | Please see our response to comment 5 above. |
| 8. The curious term "statistical evidence" means statistically significant I assume. There is a variety of statistical evidence presented across the 55 reported outcomes. Most confidence intervals are wide and compatible with negative, positive, and null effects. The authors focus on point estimates but there needs to be some recognition that most CIs are wide and that opens consideration of what we can learn from the study. The conclusion that this NHV promoted longer-term family functioning and wellbeing for women is not compatible with most of the statistical evidence. This should also include examination of differences between 4 and 5 year results - why are there effects at 4 and not 5 and vice versa? Otherwise it contributes to cherry picking those results that favour the story that the intervention 'works'. Most of the evidence presented including the Cis suggests it is very hard to know what the effects of this NHV are ages 4 and 5. | As we describe in the Methods, in complex interventions such as right@home, which target multiple improvements (in our case the broad domains of maternal and child health, wellbeing and development) it is important to measure multiple outcomes. Prioritising a single outcome as is common in clinical RCTs is likely to understate the effect of the intervention. For justification in the paper, we cite Craig P, Dieppe P, Macintyre S, Michie S, Nazareth I, Petticrew M. Developing and evaluating complex interventions: The new Medical Research Council guidance. BMJ 2008;337.  We use the term ‘statistical evidence’ to identify group differences where the confidence intervals are telling us how likely it would be that we would see a similar estimate in another trial and thus how replicable our findings are. However, we interpret the level of statistical evidence as a continuum rather than relying on the distinction of p values less than 0.05 as being statistically significant and those higher as not. To increase clarity, we have added the following to the Discussion:  “*The balance of evidence across the outcomes (as illustrated in the forest plots) indicate beneficial outcomes overall. However, a limitation of the MI/IPW methods is that they introduce uncertainty into the estimates. As such, all estimates had wide confidence intervals and few outcomes reached statistical significance, meaning it is difficult to closely identify the specific effects on each given outcome*.*”* |
| 9. What is the relevance of stating that CI were 'generally narrower" for complete case? Of course they are, but what is this alluding to? | The complete case analyses benefit from using observed data only and in this case have resulted in narrower confidence intervals. The risk is that these estimates may have been biased from the originally randomised groups on account of selective attrition from the group allocation or selective missing data. The missing data techniques add assurance that any bias has been taken account of but have added uncertainty into the interpretation. We have added the following to the Strengths section of the Discussion:  “*Consistency between the complete case analysis and analysis with missing data accounted for, indicates that the intervention benefits evident in both sets of analyses are unlikely to be artefacts*.” |
| 10. P 24 states that there was "emerging benefits' for reduced intimate partner violence. There was 1 percentage point difference at 4 and 11 points at 5 - does this mean it was "emerging"? Again this seems an overly optimistic interpretation. It would be worthwhile also stating what these same findings for intimate partner violence were at earlier ages in the previous publications. | Thank you for noting this. We have revised this section to add clarity as follows:  “When considered with the earlier waves of follow-up, this study found emerging benefits for child behaviour (which were not evident when first assessed at 3 years), and sustained benefits across parenting, family functioning and maternal mental health outcomes which increased at 5 years. *At 5 years, there was also evidence of reduced intimate partner emotional abuse*. *This is notable given that the intervention was a parenting intervention, rather than one that specifically targeted mental health or intimate partner abuse*. Given the dearth of effective preventative interventions targeting inequitable outcomes, our research strengthens the evidence for NHV as an important part of a proportionate universal healthcare system.” |
| 11. P26 end 2 nd para - a statement is made about intervention results being evident over and above potential selection bias related to more women undertaking further study in the control group. I don't know how the authors can know this. If there was selection bias on this or other characteristics it's hard to know how that affects any particular outcome. | Thank you, we have removed this comment. The revised Strengths paragraph to include:  “Recognising that NHV trials are susceptible to participant attrition, *which could introduce non-comparability between the trial arms,* we applied the best practice methods of using a combined MI/IPW approach to address missing data” |
| 12. P24 Multiple imputation and IPW weighting for attrition is not a "limitation" because it introduces "uncertainty" into estimates. There are several sources of "uncertainty" in producing any point estimate. MI/IPW is the "best" approach we have given current knowledge so this extra "uncertainty" is real and should be reflected in consideration of what we can learn from these results, given attrition, which is a feature of all such RCTs in vulnerable populations like this. The limitation is potential bias introduced by attrition - MI and IPW are the best that can be done to try to account for that. If that is the case - MI/IPW is the best we have - then why show complete case analysis at all? | Please see our response to Comment 9 above. |
| 13. Some consideration of why self-report outcomes showed some differences but not for objective assessments needs to be included. That is not uncommon in the NHV literature. | As the Reviewer suggests, the Discussion describes this finding as follows: “It is also important to consider why group differences were evident for outcomes reported by mothers but not for those that were directly assessed. Ongoing follow-up of the right@home cohort when children start school will enable comparison of teacher and parent reported data, and reveal whether developmental impacts emerge at a later age.”  We also note that the self-report measures are more common for maternal outcomes, and the direct report measures were more common for child outcomes. As per the program logic, we expect parent impacts to be evident before (and lead to) child impacts. |
